# Design, Synthesis, and Biological Evaluation of EdAP, a 4′-Ethynyl-2′-Deoxyadenosine 5′-Monophosphate Analog, as a Potent Influenza a Inhibitor

**DOI:** 10.3390/molecules24142603

**Published:** 2019-07-17

**Authors:** Toshifumi Takeuchi, Nongluk Sriwilaijaroen, Ayako Sakuraba, Ei Hayashi, Shinji Kamisuki, Yasuo Suzuki, Hiroshi Ohrui, Fumio Sugawara

**Affiliations:** 1 Department of Applied Biological Science, Faculty of Science and Technology, Tokyo University of Science, 2641 Yamazaki, Noda, Chiba 278-8510, Japan; 2 Department of Preclinical Sciences, Faculty of Medicine, Thammasat University, Pathumthani 12120, Thailand; 3 Health Science Hills, College of Life and Health Sciences, Chubu University, Kasugai, Aichi 487-8501, Japan; 4 School of Veterinary Medicine, Azabu University, 1-17-71 Fuchinobe, Chuo-ku, Sagamihara, Kanagawa 252-5201, Japan; 5 Yokohama University of Pharmacy, Matano-cho 601, Totsuka-ku, Yokohama, Kanagawa 245-0066, Japan

**Keywords:** 4′-ethynyl-2′-deoxyadenosine 5′-monophosphate, influenza, anti-influenza activity

## Abstract

Influenza A viruses leading to infectious respiratory diseases cause seasonal epidemics and sometimes periodic global pandemics. Viral polymerase is an attractive target in inhibiting viral replication, and 4′-ethynyladenosine, which has been reported as a highly potent anti-human immunodeficiency virus (HIV) nucleoside derivative, can work as an anti-influenza agent. Herein, we designed and synthesized a 4′-ethynyl-2′-deoxyadenosine 5′-monophosphate analog called EdAP (**5**). EdAP exhibited potent inhibition against influenza virus multiplication in Madin–Darby canine kidney (MDCK) cells transfected with human α2-6-sialyltransferase (SIAT1) cDNA and did not show any toxicity toward the cells. Surprisingly, this DNA-type nucleic acid analog (**5**) inhibited the multiplication of influenza A virus, although influenza virus is an RNA virus that does not generate DNA.

## 1. Introduction

Influenza caused by influenza A virus is an infectious respiratory disease, which is responsible for seasonal epidemics and periodic global pandemics. This infection causes mild to severe illness, including death, in persons with an increased risk for severe diseases, including the elderly, infants, and the ill, resulting in three to five million severe cases and 250,000 to 500,000 deaths worldwide [1]. The most efficient prevention method in healthy adults is vaccination. However, for the elderly, vaccination may be less efficient and may increase complications and death. Four classes of antiviral medicines are currently available. These include (i) the neuraminidase inhibitors oseltamivir (Roche), zanamivir (GlaxoSmithKline), peramivir (BioCryst Pharmaceuticals), and laninamivir octanoate (Daiichi Sankyo); (ii) the M2 ion channel blockers rimantadine (Sun Pharma) and amantadine (Endo); (iii) favipiravir (Toyama Chemical), which inhibits the ribonucleic acid (RNA)-dependent RNA polymerase; and (iv) a cap-dependent endonuclease inhibitor, baloxariv marboxil (Shionogi) [2,3,4]. M2 ion channel blockers have been previously used to treat influenza. However, the virus resistance of this class of medicine has been frequently reported to limit treatment efficiency.

The influenza A virus is a negative-sense single-strand RNA virus with a lipid envelope. The viral genome is divided into eight segments that encode nine structural proteins and a variable number of nonstructural proteins depending on the virus strain and host species. Each segment is packaged in a complex together with an RNA-dependent RNA polymerase, composed of polymerase basic 1 (PB1), polymerase basic 2 (PB2) and polymerase acidic (PA) proteins, involved in viral RNA transcription and replication [2].

**Figure 1:**
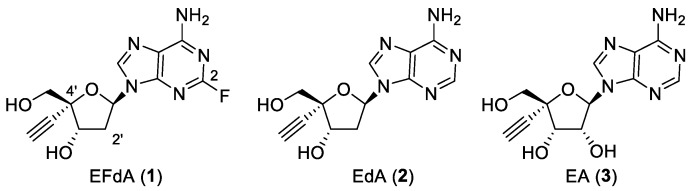
Structures of 4′-ethynyl-2-fluoro-2′-deoxyadenosine (EFdA) (**1**), 4′-ethynyl-2′-deoxyadenosine (EdA) (**2**), and 4′-ethynyl-2-fluoroadenosine (EA) (**3**).

Each viral polymerase is an attractive target for developing a new antiviral compound. The inhibitors should work against several subtypes of influenza A viruses and be tolerated by a drug-resistant mutant because it is highly conserved in all influenza subtypes and is essential to the viral life cycle. We have previously reported on a highly potent antihuman immunodeficiency virus (HIV) nucleoside derivative called 4′-ethynyl-2-fluoro-2′-deoxyadenosine (EFdA, **1**, Figure 1) [5,6,7,8,9,10,11,12,13]. EFdA is an adenosine derivative armed with an ethynyl group at the 4′-position, which prevents the extension of a nucleotide at 3′-OH because of its steric hindrance by the 4′-substituent, providing features of a viral DNA chain terminator and a reverse transcriptase inhibitor [14,15,16,17,18]. EFdA is currently used in human clinical trials [19,20,21,22,23,24]. Herein, we hypothesize that EFdA derivatives may work as an anti-influenza inhibitor. We subsequently describe the discovery of a new 4′-ethynyl-2′-deoxyadenosine 5′-monophosphate analog called EdAP (**5**) as a potent inhibitor of influenza A virus. Interestingly, this DNA-type nucleic acid analog inhibited the multiplication of influenza A virus, although influenza A virus is an RNA virus that does not generate DNA.

## 2. Results and Discussion

### 2.1. Effect of Compounds **1**–**3** on Anti-Influenza Activities

We intended to analyze the anti-influenza activity of 4′-ethynyl adenosine derivatives, namely EFdA (**1**), 4′-ethynyl-2′-deoxyadenosine (EdA, **2**) [13], and 4′-ethynyl-2-fluoroadenosine (EA, **3**), based on our hypothesis that EFdA analogs may work against the influenza virus [25]. The adenosine derivatives of compounds **1**–**3** were evaluated for their in vitro anti-influenza activities against the H1N1 virus using MDCK-SIAT1 according to a procedure that has been previously described [26]. The deoxynucleoside-type derivatives **1** and **2** displayed modest and weak activities, respectively, whereas the nucleoside-type derivative **3** did not show any activity (Table 1). The cytotoxicity of compounds **1**–**3** was also determined using a cell counting kit-8 (CCK8) assay, and the following table presents the results. No cytotoxicity was observed at the highest concentration tested by the CCK8 assay (1 mM).

**Figure 2:**
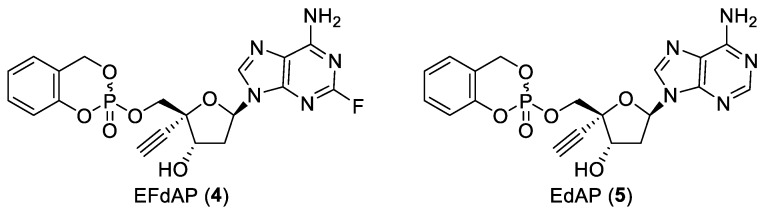
Structures of EFdAP (**4**) and EdAP (**5**).

Here, 5′-OH should have been triphosphorylated when derivatives **1** and **2** worked as a chain terminator in cells. This phosphorylation process was stepwise. First, 5′-OH was transformed by cellular phosphatase into generating 5′-*O*-monophosphate. Next, 5′-monophosthate was transformed into 5′-diphosphate and then into 5′-triphosphate. We assumed that the cellular phosphatase involved in the first step could slowly phosphorylate the 5′-OH of the derivatives because 5′-OH was sterically hindered by the 4′-ethynyl group. However, a 5′-*O*-monophosphate analog could be much more efficiently phosphorylated by cellular phosphatase to generate a 5′-diphosphate analog. We designed compounds **4** and **5** based on these assumptions (Figure 2) [27]. Compounds **4** and **5** equipped the cyclosaligenyl (cycloSal) group on phosphate ester, which increased the compound permeability [28,29,30]. This phosphate ester should have been cleaved by esterase in cells as a prodrug. We synthesized a 4′-ethynyl-2-fluoro-2′-deoxyadenosine 5′-monophosphate analog called EFdAP (**4**, Figure 2) (data not shown) and evaluated its anti-influenza activity. However, the activity (IC_50_ = 57 μM) was not improved when compared to EFdA. Therefore, we decided to synthesize and evaluate the activity of EdAP (**5**).

### 2.2. Synthesis of EdAP (**5**)

**Scheme 1:**
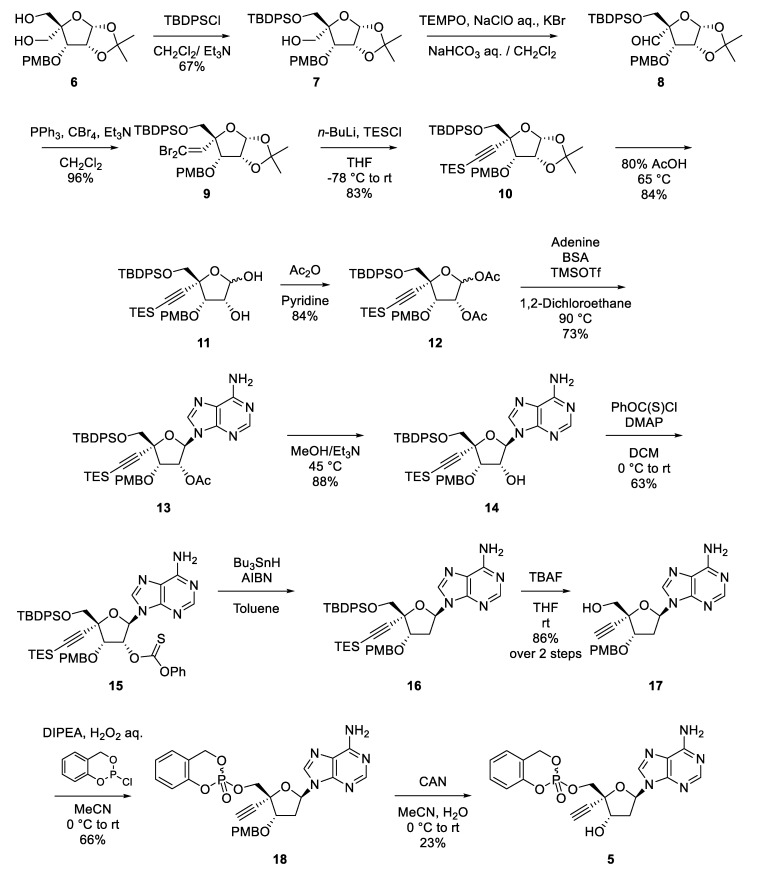
Synthesis of EdAP (**5**).

The synthesis of derivative **5** was initiated from diol **6** [31] (Scheme 1). The *tert*-butyldiphenylsilyl (TBDPS) protection of β-methylene alcohol in **6** generated alcohol **7** in a 67% yield. The (2,2,6,6-tetramethylpiperidin-1-yl)oxyl (TEMPO) oxidation of alcohol **7** and the subsequent treatment of the resulting aldehyde **8** with tetrabromomethane and triphenylphosphine generated dibromoolefine **9** in a 96% yield over two steps [32]. Dibromoolefine **9** was treated with *n*-BuLi, followed by chlorotriethylsilane(TESCl), to provide chlorotriethylsilane (TES)-protected alkyne **10** in an 83% yield. The treatment of the resulting acetal **10** with AcOH generated hemiacetal **11**, which was acetylated with Ac_2_O to afford diacetate **12** as a diastereomeric mixture of the acetoxy group in an 84% yield. The one-pot Silyl–Hilbert–Johnson reaction between adenine and acetate **12** furnished the desired adenosine derivative **13** in a 73% yield [33,34]. After the deacetylation of **13**, the resulting alcohol **14** was converted to phenylthiocarbonate **15** in a 63% yield, which was treated with Bu_3_SnH and azobisisobutyronitrile (AIBN) to generate deoxy adenosine derivative **16** in an 86% yield [35,36]. The deprotection of the TBDPS group in **16** and the introduction of phosphate ester by chlorophosphite **17** and H_2_O_2_ aq, followed by the deprotection of the *p*-Methoxybenzyl (PMB) group by cerium ammonium nitrate (CAN), afforded EdAP (**5**).

### 2.3. Anti-Influenza Activity of EdAP (**5**)

**Figure 3:**
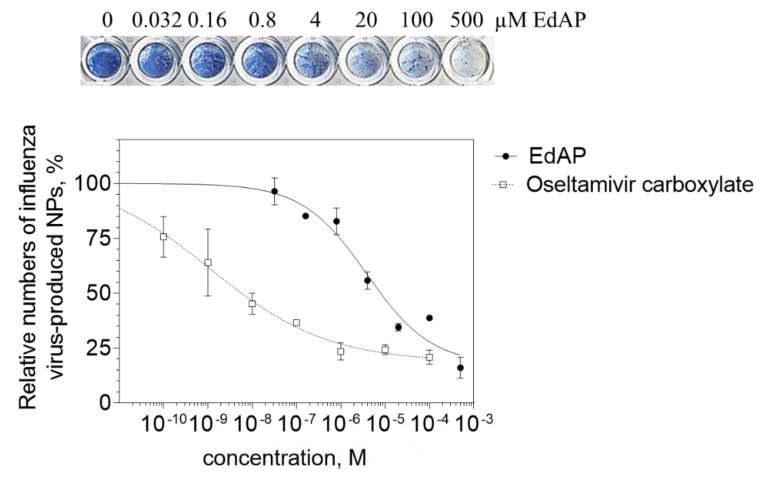
Inhibition of the influenza virus growth in the MDCK–SIAT1 cells by EdAP (**5**). Upper panel: The number of influenza virus-produced nucleoproteins (NPs) in cells decreased with an increase in EdAP concentration, as indicated. Lower panel: The reduction in the relative numbers of influenza virus-produced NPs in cells was a function of EdAP (filled circle and solid line) or oseltamivir carboxylate (open square and dashed line) concentrations, as indicated.

The in vitro anti-influenza activity of compound **5** was analyzed (Figure 3). Consequently, compound **5** significantly inhibited the growth of the influenza virus, with an IC_50_ of 3.8 μM, whereas no cytotoxicity was observed at the highest concentration tested by the CCK8 assay (1 mM). The maximum inhibition level of **5** was around a 79% decrease of influenza virus-produced NPs. This level is comparable to that of oseltamivir. Considering the result that the anti-influenza activity of the parent compound **2** was weak, phosphate ester **5** presumably worked as a prodrug. The introduced phosphate ester could be cleaved in cells to generate phosphate monoester. EdA (**2**) was easily hydrolyzed by adenosine deaminase to generate the corresponding inactive inosine derivative [6,11,37]. However, EdAP, which has the same scaffold as EdA, except in the case of cyclosaligenyl phosphate ester, exhibited anti-influenza activity. Thus, the cycloSal phosphate group could prevent the conversion of EdAP by adenosine deaminase, or other unknown mechanisms may exist.

## 3. Materials and Methods

### 3.1. Chemistry

Unless otherwise indicated, all the reactions were performed in oven-dried glassware fitted with a 3-way glass stopcock under an argon atmosphere and stirred with Teflon-coated magnetic stir bars. All work-up and purification procedures were performed with reagent-grade solvents under an ambient atmosphere. CH_2_Cl_2_ was distilled from P_2_O_5_ immediately before use. Et_2_O and tetrahydrofuran (THF) were distilled from sodium/benzophenone immediately before use. Et_3_N and *N*,*N*-diisopropylethylamine (DIEPA) were distilled from CaH_2_ and stored over KOH. CH_3_CN and toluene were distilled from CaH_2_ and stored over molecular sieves 4A. Chemical regents were of commercial grade and were used without any purification unless otherwise noted. Flash chromatography was performed on a PSQ-100B (Fuji Silysia Co., Ltd., Aichi, Japan) unless otherwise noted. Analysis thin layer chromatography (TLC) was performed using commercial silica gel plates (TLC Silica Gel 60 F_254_, Merck Millipore, Massachusetts, United States). Infrared spectra (IR) were recorded on a Jasco FT/IR-410 spectrometer using NaCl (neat). High-resolution mass spectra (HRMS) were obtained from an Applied Biosystems mass spectrometer (APIQSTAR pulsar I) for electrospray ionization (ESI). Polyethylene glycol was used as the internal standard. HRMS data are reported as *m*/*z* (relative intensity), with an accurate mass reported for the molecular ion [M + Na]^+^. ^1^H and ^13^C-NMR spectra were recorded on a Bruker 600 MHz spectrometer (Avance DRX-600) with CDCl_3_ or MeOD as a solvent. Tetramethylsilane (TMS) was used as an internal standard (δ 0.0) for ^1^H-NMR. Chemical shifts for ^13^C-NMR were reported in ppm relative to the center line of a triplet at 77.0 ppm in CDCl_3_ or a septet at 49.0 ppm in MeOD as a solvent. Multiplicities are indicated as br (broad), s (singlet), d (doublet), t (triplet), q (quartet), or m (multiplet). Coupling constants (*J*) are reported in hertz (Hz). The diastereomeric ratio was determined by ^1^H-NMR analysis. Optical rotations were determined using a JASCO P-1010 digital polarimeter in 100-mm cells and with a sodium D line (589 nm) at room temperature in the solvent and concentration indicated.

#### 3.1.1. Synthesis of ((3a*R*,5*R*,6*S*,6a*R*)-5-(((*tert*-butyldiphenylsilyl)oxy)methyl)-6-((4-methoxybenzyl)oxy)-2,2-dimethyltetrahydrofuro [2,3-*d*][1,3]dioxol-5-yl)methanol (**7**)

To a stirred solution of diol **6** (10.3 g, 30 mmol) in CH_2_Cl_2_ (40 mL) and Et_3_N (20 mL) was added *tert*-butylchlorodiphenylsilane (7.1 mL, 27 mmol). After stirring for 16 h, saturated aqueous NaCO_3_ solution was added to the mixture. The resulting reaction mixture was extracted with EtOAc, and the combined organic layer was washed with H_2_O, dried over brine and Na_2_SO_4_, and concentrated under reduced pressure. The resulting residue was purified by silica gel column chromatography to afford alcohol **7** (10.9 g, 63%) as a white crystal. **7**: Colorless crystal; *R*_f_ 0.8 (hexane/EtOAc = 1/1); [α]_D_^20^ + 38.1 (*c* 1.15, CHCl_3_); ^1^H-NMR (600 MHz, CDCl_3_), *δ* in ppm 7.63–7.59 (m, 4H), 7.44–7.40 (m, 2H), 7.38–7.35 (m, 4H), 7.28 (d, *J* = 8.6 Hz, 2H), 6.86 (d, *J* = 8.6 Hz, 2H), 5.81 (d, *J* = 3.8 Hz, 1H), 4.75 (d, *J* = 11.6 Hz, 1H), 4.67 (dd, *J* = 5.2, 3.8 Hz, 1H), 4.45 (d, *J* = 11.6 Hz, 1H), 4.42 (d, *J* = 5.2 Hz, 1H), 3.81 (dd, *J* = 11.9, 6.4 Hz, 1H), 3.79 (dd, *J* = 11.9, 7.2 Hz, 1H), 3.79 (s, 3H), 3.75 (d, *J* = 11.1 Hz, 1H), 3.66 (d, *J* = 11.1 Hz, 1H), 2.41 (dd, *J* = 7.2, 6.4 Hz, 1H), 1.65 (s, 3H), 1.37 (s, 3H), 0.99 (s, 9H); ^13^C-NMR (150 MHz, CDCl_3_) δ_C_ 159.5, 135.60 (2C), 135.55 (2C), 133.2, 133.0, 129.74, 129.67, 129.5 (2C), 129.3, 127.71 (2C), 127.68 (2C), 114.0 (2C), 113.7, 104.5, 87.4, 79.2, 77.5, 72.2, 65.4, 63.3, 55.2, 26.9, 26.8 (3C), 26.3, 19.2; IR ν_max_ (film) 3545, 2939, 2862, 1612, 1514, 1464, 1429, 1379, 1250, 1215, 1107, 1028 cm^−1^; HRMS (ESI) *m*/*z* = calcd 601.2592 [M + Na]^+^, found 601.2600 [M + Na]^+^; mp 113 °C (see Supplementary Materials).

#### 3.1.2. Synthesis of tert-butyl(((3aR,5R,6S,6aR)-5-(2,2-dibromovinyl)-6-((4-methoxybenzyl)oxy)-2,2- dimethyltetrahydrofuro[2,3-d][1,3]dioxol-5-yl)methoxy)diphenylsilane (**9**)

To a stirred solution of **7** (17.4 g, 30 mmol) in CH_2_Cl_2_ (60 mL) and NaHCO_3_ (60 mL, 0.25 M in H_2_O) were added TEMPO (47 mg, 0.3 mmol) and KBr (360 mg, 3.0 mmol). After the slow addition of NaOCl (24 mL, 12% in H_2_O) at 0 °C, the resulting mixture was stirred for 30 min. The resulting reaction mixture was extracted with EtOAc, and the combined organic layer was washed with H_2_O, dried over brine and Na_2_SO_4_, and concentrated under reduced pressure to give crude aldehyde **8**, which was directly used in the next step without further purification. To a stirred solution of the above aldehyde **8** in CH_2_Cl_2_ (100 mL) and Et_3_N (16.7 mL, 120 mmol) were added CBr_4_ (20 g, 60 mmol) and PPh_3_ (31.5 g, 120 mmol) at 0 °C. After stirring for 18 h at room temperature (rt), the reaction was quenched with H_2_O. After filtration of the resulting reaction mixture through a pad of celite, the filtrate was concentrated under reduced pressure. The resulting residue was purified by silica gel column chromatography to afford dibromoolefine **9** (21 g, 96%) as a colorless oil. **9**: Pale yellow oil; *R*_f_ 0.6 (hexane/EtOAc = 4/1); [α]_D_^19^ −40.1 (*c* 1.02, CHCl_3_); ^1^H-NMR (600 MHz, CDCl_3_), δ in ppm 7.66–7.61 (m, 4H), 7.44–7.30 (m, 8H), 7.08 (s, 1H), 6.86 (d, *J* = 8.6 Hz, 2H), 5.73 (d, *J* = 4.0 Hz, 1H), 4.75 (d, *J* = 11.9 Hz, 1H), 4.62 (d, *J* = 11.9 Hz, 1H), 4.55 (dd, *J* = 4.3, 4.0, 1H), 4.35 (d, *J* = 4.3 Hz, 1H), 3.99 (d, *J* = 11.6 Hz, 1H), 3.77 (s, 3H), 3.54 (d, *J* = 11.6, 1H), 1.58 (s, 3H), 1.32 (s, 3H), 0.98 (s, 9H); ^13^C-NMR (150 MHz, CDCl_3_) δ_C_ 170.9, 159.5, 135.9 (2C), 135.5 (2C), 135.3, 133.5, 132.6, 129.7 (2C), 129.6 (2C), 129.4, 127.7 (2C), 127.6 (2C), 114.0 (2C), 113.1, 103.8, 90.5, 85.6, 76.1, 72.2, 61.6, 55.2, 26.8 (3C), 26.0, 25.8, 19.3; IR ν_max_ (film) 3047, 2862, 1608, 1514, 1252, 1107, 1024 cm^−1^; HRMS (ESI) *m*/*z* = calcd 753.0853 [M + Na]^+^, found 753.0877 [M + Na]^+^.

#### 3.1.3. Synthesis of tert-butyl(((3aR,5R,6S,6aR)-6-((4-methoxybenzyl)oxy)-2,2-dimethyl-5-((triethylsilyl) ethynyl)tetrahydrofuro[2,3-d][1,3]dioxol-5-yl)methoxy)diphenylsilane (**10**)

To a stirred solution of dibromoalkene **9** (21 g, 29 mmol) in THF (300 mL) was added *n*-BuLi (20.9 mL, 2.76 M in hexane) at −78 °C. After stirring for 2 h at −30 °C, *n*-BuLi (4.2 mL, 2.76 M in hexane) was added. After stirring for 1 h at −30 °C, chlorotriethylsilane (5 mL, 30 mmol) was added at −78 °C. After stirring for 2 h at −78 °C, the reaction was quenched with H_2_O at 0 °C. The resulting mixture was extracted with EtOAc, and the combined organic layer was washed with H_2_O, dried over brine and Na_2_SO_4_, and concentrated under reduced pressure. The resulting residue was purified by silica gel column chromatography to afford acetonide **10** (16.5 g, 83%) as a colorless oil. **10**: Pale yellow oil; *R*_f_ 0.7 (hexane/EtOAc = 4/1); [α]_D_^22^ -49.0 (*c* 0.52, CHCl_3_); ^1^H-NMR (600 MHz, CDCl_3_), δ in ppm 7.63–7.60 (m, 4H), 7.44–7.35 (m, 6H), 7.31 (d, *J* = 8.6 Hz, 2H), 6.83 (d, *J* = 8.6 Hz, 2H), 5.73 (d, *J* = 3.5 Hz, 1H), 4.70–4.64 (m, 3H), 4.41 (d, *J* = 4.7 Hz, 1H), 3.91 (d, *J* = 11.3 Hz, 1H), 3.78 (s, 3H), 3.70 (d, *J* = 11.3 Hz, 1H), 1.74 (s, 3H), 1.36 (s, 3H), 0.97 (s, 9H), 0.93 (t, *J* = 7.9 Hz, 9H), 0.56 (q, *J* = 7.9 Hz, 6H); ^13^C-NMR (150 MHz, CDCl_3_) δ_C_ 159.2, 135.7 (2C), 135.5 (2C), 133.3, 132.7, 130.0, 129.73, 129.69, 129.2 (2C), 127.71 (2C), 127.70 (2C), 114.1, 113.7 (2C), 103.9, 103.4, 90.9, 81.9, 79.5, 76.4, 71.9, 67.3, 55.2, 26.8 (3C), 26.5, 26.4, 19.3, 7.4 (3C), 4.1 (3C); IR ν_max_ (film) 2952, 2166, 1514, 1462, 1167 cm^-1^; HRMS (ESI) *m*/*z* = calcd 709.3351 [M + Na]^+^, found 709.3358 [M + Na]^+^.

#### 3.1.4. Synthesis of (3R,4S,5R)-5-(((tert-butyldiphenylsilyl)oxy)methyl)-4-((4-methoxybenzyl)oxy)-5- ((triethylsilyl)ethynyl)tetrahydrofuran-2,3-diyl diacetate (**12**)

A solution of acetonide **10** (16.5 g, 24 mmol) in AcOH (240 mL) and H_2_O (60 mL) was stirred for 5 h at 65 °C. Concentration of the reaction mixture with toluene gave crude hemiacetal **11**, which was directly used in the next step without further purification. A solution of the above crude hemiacetal **11** in pyridine (60 mL) and Ac_2_O (20 mL) was stirred for 10 h at rt. After concentration of the reaction mixture with toluene, the resulting residue was purified by silica gel column chromatography to afford acetal **12** (14.5 g, 83%) as a colorless oil. **12**: Pale yellow oil; *R*_f_ 0.5 (hexane/EtOAc = 4/1); ^1^H-NMR (600 MHz, CHCl_3_), δ in ppm 7.67–7.62 (m, 4H), 7.44–7.40 (m, 2H), 7.38–7.35 (m, 4H), 7.24 (d, *J* = 8.6 Hz, 2H), 6.84 (d, *J* = 8.6 Hz, 2H), 6.28 (s, 1H), 5.36 (d, *J* = 4.9 Hz, 1H), 4.60–4.55 (3H, overlapped), 3.85 (d, *J* = 11.1 Hz, 1H), 3.79 (s, 3H), 3.77 (d, *J* = 11.1 Hz, 1H), 2.09 (s, 3H), 1.80 (s, 3H), 1.05 (s, 9H), 0.92 (t, *J* = 7.9 Hz, 9H), 0.55 (q, *J* = 7.9 Hz, 6H); HRMS (ESI) *m*/*z* = calcd 753.3249 [M + Na]^+^, found 753.3259 [M + Na]^+^.

#### 3.1.5. Synthesis of (2R,3R,4S,5R)-2-(6-amino-9H-purin-9-yl)-5-(((tert-butyldiphenylsilyl)oxy)methyl)-4- ((4-methoxybenzyl)oxy)-5-((triethylsilyl)ethynyl)tetrahydrofuran-3-yl acetate (**13**)

To a stirred solution of acetal **12** (5.7 g, 7.8 mmol) in 1,2-dichloroethane (15 mL) were added adenine (3.2 g, 24 mmol) and *N*,*O*-bis(trimethylsilyl)acetamide (11.5 mL, 47 mmol). After stirring for 5 h under reflux conditions, trimethylsilyl trifluoromethanesulfonate (TMSOTf) (4.2 mL, 23 mmol) was added at 0 °C. After stirring for 10 h under reflux conditions, a saturated NaHCO_3_ solution was added to the mixture at rt. After filtration of the resulting mixture through a pad of celite, the filtrate was extracted with EtOAc, and the combined organic layer was washed with H_2_O, dried over brine and Na_2_SO_4_, and concentrated under reduced pressure. The resulting residue was purified by silica gel column chromatography to afford nucleoside **13** (4.6 g, 73%) as a colorless oil. **13**: Colorless oil; *R*_f_ 0.3 (Hexane/EtOAc = 1/2); [α]_D_^20^ -31.3 (*c* 0.61, CHCl_3_); ^1^H-NMR (600 MHz, CDCl_3_), δ in ppm 8.19 (s, 1H), 7.91 (s, 1H), 7.65–7.62 (m, 4H), 7.45–7.36 (m, 2H), 7.34–7.32 (m, 4H), 7.27 (d, *J* = 8.5 Hz, 2H), 6.85 (d, *J* = 8.5 Hz, 2H), 6.28 (d, *J* = 5.0 Hz, 1H), 5.93 (1H, dd, *J* = 6.1, 5.0 Hz, 1H), 5.47 (brs, 2H), 4.82 (d, *J* = 6.1 Hz, 1H), 4.73 (d, *J* = 11.1 Hz, 1H), 4.54 (d, *J* = 11.1 Hz, 1H), 4.03 (d, *J* = 11.2 Hz, 1H), 3.81 (d, *J* = 11.2 Hz, 1H), 3.80 (s, 3H), 2.02 (s, 3H), 1.03 (s, 9H), 0.96 (t, *J* = 7.9 Hz, 9H), 0.59 (q, *J* = 7.9 Hz, 6H); ^13^C-NMR (150 MHz, CDCl_3_) δ_C_ 170.0, 159.2, 155.3, 153.2, 149.8, 139.7, 135.6 (2C), 135.5 (2C), 132.64, 132.56, 129.89, 129.86, 129.82, 129.3 (2C), 127.7 (4C), 120.1, 113.6 (2C), 102.0, 91.6, 86.5, 83.5, 76.5, 73.6, 73.3, 66.7, 55.3, 26.8 (3C), 20.6, 19.2, 7.4 (3C), 4.2 (3C); IR ν_max_ (film) 3319, 3167, 2170, 1745, 1599, 1469 cm^−1^; HRMS (ESI) *m*/*z* = calcd 828.3583 [M + Na]^+^, found 828.3559 [M + Na]^+^.

#### 3.1.6. Synthesis of (2R,3R,4S,5R)-2-(6-amino-9H-purin-9-yl)-5-(((tert-butyldiphenylsilyl)oxy)methyl)-4-((4- methoxybenzyl)oxy)-5-((triethylsilyl)ethynyl)tetrahydrofuran-3-ol (**14**)

A solution of nucleoside **13** (4.6 g, 5.7 mmol) in MeOH (60 mL) and Et_3_N (15 mL) was stirred for 18 h at rt. After concentration of the reaction mixture, the resulting residue was purified by silica gel column chromatography to afford alcohol **14** (3.5 g, 80%) as a colorless oil. **14**: Colorless oil; *R*_f_ 0.5 (EtOAc); [α]_D_^21^ −19.8 (*c* 0.66, CDCl_3_); ^1^H-NMR (600 MHz, CHCl_3_), δ in ppm 8.20 (s, 1H), 7.90 (s, 1H), 7.62–7.60 (m, 4H), 7.43–7.40 (m, 2H), 7.34–7.32 (m, 6H), 6.88 (d, *J* = 8.6 Hz, 2H), 6.07 (d, *J* = 5.1 Hz, 1H), 5.48 (br s, 2H), 4.91 (d, *J* = 11.2 Hz, 1H), 4.87 (ddd, *J* = 7.9, 6.1, 5.1 Hz, 1H), 4.67 (d, *J* = 11.2 Hz, 1H), 4.58 (d, *J* = 6.1 Hz, 1H), 3.99 (d, *J* = 11.0 Hz, 1H), 3.83 (d, *J* = 11.0 Hz, 1H), 3.81 (s, 3H), 3.56 (d, *J* = 7.9 Hz, 1H), 1.02 (s, 9H), 0.97 (t, *J* = 7.9 Hz, 9H), 0.62 (q, *J* = 7.9 Hz, 6H); ^13^C-NMR (150 MHz, CDCl3) δ_C_ 159.6, 155.3, 153.0, 149.7, 139.8, 135.6 (2C), 135.5 (2C), 132.6, 132.5, 129.92, 129.89, 129.7 (2C), 129.2, 127.78 (2C), 127.76 (2C), 120.2, 113.9 (2C), 102.5, 92.1, 89.9, 83.3, 77.3, 73.2, 73.0, 66.9, 55.3, 26.8 (3C), 19.2, 7.4 (3C), 4.2 (3C); IR ν_max_ (film) 3327, 3172, 2170, 1645, 1471, 1109 cm^−1^; HRMS (ESI) *m*/*z* = calcd 786.3482 [M + Na]^+^, found 786.3499 [M + Na]^+^.

#### 3.1.7. Synthesis of *O*-((2R,3R,4S,5R)-2-(6-amino-9H-purin-9-yl)-5-(((tert-butyldiphenylsilyl)oxy)methyl)-4- ((4-methoxybenzyl)oxy)-5-((triethylsilyl)ethynyl)tetrahydrofuran-3-yl) *O*-phenyl carbonothioate (**15**)

To a stirred solution of alcohol **14** (3.5 g, 4.6 mmol) in MeCN (30 mL) were added *N*,*N*-dimethyl-4-aminopyridine (DMAP) (1.1 g, 9.2 mmol) and *O*-phenyl carbonochloridothioate (635 μL, 4.6 mmol) at 0 °C. After stirring for 21 h, the reaction mixture was concentrated under reduced pressure. The resulting residue was purified by silica gel column chromatography to afford thiocarbonate **15** (2.6 g, 63%) as a colorless oil. **15**: Colorless oil; *R*_f_ 0.6 (hexane/EtOAc = 1/2); [α]_D_^21^ −63.8 (*c* 0.52, CHCl_3_); ^1^H-NMR (600 MHz, CDCl_3_), δ in ppm 8.15 (s, 1H), 7.91 (s, 1H), 7.67–7.63 (m, 4H), 7.43-7.39 (m, 2H), 7.37–7.28 (m, 9H), 6.91 (d, *J* = 8.3 Hz, 2H), 6.87 (d, *J* = 8.7 Hz, 2H), 6.47 (dd, *J* = 5.9, 5.3 Hz, 1H), 6.41 (d, *J* = 5.3 Hz, 1H), 5.49 (brs, 2H), 5.11 (d, *J* = 5.9 Hz, 1H), 4.87 (d, *J* = 11.0 Hz, 1H), 4.64 (d, *J* = 11.0 Hz, 1H), 4.11 (d, *J* = 11.2 Hz, 1H), 3.86 (d, *J* = 11.2 Hz, 1H), 3.81 (s, 3H), 1.03 (s, 9H), 0.97 (t, *J* = 7.9 Hz, 9H), 0.61 (q, *J* = 7.9 Hz, 6H); ^13^C-NMR (150 MHz, CDCl_3_) δ_C_ 194.3, 159.3, 155.3, 153.4, 153.3, 149.9, 140.1, 135.64 (2C), 135.61 (2C), 132.6 (2C), 129.9, 129.81, 129.80, 129.6 (2C), 129.5 (2C), 127.75 (2C), 127.73 (2C), 126.6, 121.7 (2C), 120.2, 113.7 (2C), 101.8, 92.2, 86.2, 83.6, 80.6, 76.2, 73.8, 66.6, 55.3, 26.8 (3C), 19.2, 7.4 (3C), 4.2 (3C); IR ν_max_ (film) 3315, 2956, 2171, 1643, 1105, 1009 cm^−1^; HRMS (ESI) *m*/*z* = calcd 922.3460 [M + Na]^+^, found 922.3451 [M + Na]^+^.

#### 3.1.8. Synthesis of 9-((2R,4S,5R)-5-(((tert-butyldiphenylsilyl)oxy)methyl)-4-((4-methoxybenzyl)oxy)-5-((triethylsilyl)ethynyl)tetrahydrofuran-2-yl)-9H-purin-6-amine (**17**)

To a stirred solution of thiocarbonate **15** (2.6 g, 2.9 mmol) in toluene (60 mL) were added HSnBu_3_ (4.6 mL, 17 mmol) and AIBN (117 mg, 0.7 mmol). After stirring for 30 min at 130 °C, the reaction mixture was concentrated under reduced pressure. The resulting residue was roughly purified by silica gel column chromatography to afford nucleoside **16** as a colorless oil. To a stirred solution of nucleoside **16** above in THF (20 mL) was added tetra-*n*-butylammonium fluoride (TBAF) (4.8 mL, 1 M in THF) at rt. After stirring for 2 h, the reaction mixture was concentrated under reduced pressure. The resulting residue was purified by silica gel column chromatography to afford alcohol **17** (982 mg, 86%) as a colorless oil. **17**: Pale yellow crystal; *R*_f_ 0.5 (EtOAc); [α]_D_^21^ + 6.41 (*c* 0.56, CHCl_3_); ^1^H-NMR (600 MHz, CDCl_3_), δ in ppm 8.30 (s, 1H), 7.77 (s, 1H), 7.35 (d, *J* = 8.4 Hz, 2H), 6.90 (d, *J* = 8.4 Hz, 2H), 6.80 (br d, *J* = 12.3 Hz, 1H), 6.36 (dd, *J* = 9.2, 5.7 Hz, 1H), 5.76 (brs, 2H), 4.82 (d, *J* = 11.3 Hz, 1H), 4.60 (d, *J* = 11.3 Hz, 1H), 4.52 (d, *J* = 5.8 Hz, 1H), 4.06 (d, *J* = 12.5 Hz, 1H), 3.84 (dd, *J* = 12.5, 12.3 Hz, 1H), 3.82 (s, 3H), 3.09 (ddd, *J* = 13.5, 9.2, 5.8 Hz, 1H), 2.72 (s, 1H), 2.44 (dd, *J* = 13.5, 5.7 Hz, 1H); ^13^C-NMR (150 MHz, CDCl_3_) δ_C_ 159.4, 155.9, 152.4, 148.5, 140.2, 129.6, 129.4 (2C), 121.2, 113.9 (2C), 87.2, 86.7, 80.3, 79.7, 77.0, 72.5, 68.6, 55.3, 38.8; IR ν_max_ (film) 3298, 2949, 2400, 1682, 1514, 1425 cm^−1^; HRMS (ESI) *m*/*z* = calcd 418.1485 [M + Na]^+^, found 418.1488 [M + Na]^+^.

#### 3.1.9. Synthesis of 2-(((2R,3S,5R)-5-(6-amino-9H-purin-9-yl)-2-ethynyl-3-((4-methoxybenzyl)oxy) tetrahydrofuran-2-yl)methoxy)-4H-benzo[d][1,3,2]dioxaphosphinine 2-oxide (**18**)

To a stirred solution of alcohol **17** (200 mg, 0.51 mmol) in MeCN (5 mL) were added DIPEA (178 μL, 1.0 mmol) and 2-chloro-4*H*-benzo[d][1,3,2]dioxaphosphinine (192 mg, 0.51 mmol) at 0 °C. After stirring for 1 h at 0 °C, H_2_O_2_ (250 μL, 30% in H_2_O) was added at 0 °C. After stirring for 1 h at 0 °C, the resulting mixture was directly purified by silica gel column chromatography to afford phosphoester **18** (220 mg, 76%) as a colorless oil. **18**: Pale yellow oil; *R*_f_ 0.7 (CHCl_3_/MeOH = 8/1); [α]_D_^21^ −7.85 (*c* 0.53, CHCl3); ^1^H-NMR (600 MHz, CDCl_3_), δ in ppm major-isomer 8.19 (s, 1H), 7.78 (s, 1H), 7.32–7.24 (4H, overlapped), 7.12–7.09 (m, 1H), 6.99–6.94 (2H, overlapped), 6.90–6.86 (2H, overlapped), 6.34–6.31 (1H, overlapped), 5.60 (2H, brs), 5.26–5.14 (2H, overlapped), 4.72–4.67 (2H, overlapped), 4.58 (d, *J* = 11.6 Hz, 1H), 4.52–4.49 (m, 1H), 4.37–4.34 (m, 1H), 3.80 (s, 3H), 2.89–2.85 (1H, overlapped), 2.67 (s, 1H), 2.66–2.61 (1H, overlapped); minor-isomer 8.26 (s, 1H), 7.80 (s, 1H), 7.32–7.24 (4H, overlapped), 7.09–7.06 (m, 1H), 6.99–6.94 (2H, overlapped), 6.90–6.86 (2H, overlapped), 6.34–6.31 (1H, overlapped), 5.62 (2H, brs), 5.26–5.14 (2H, overlapped), 4.72–4.67 (2H, overlapped), 4.62 (d, *J* = 11.6 Hz, 1H), 4.47–4.44 (m, 1H), 4.41–4.38 (m, 1H), 3.81 (s, 3H), 2.89–2.85 (1H, overlapped), 2.67 (s, 1H), 2.66–2.61 (1H, overlapped); IR ν_max_ (film) 3175, 2114, 1643, 1597, 1513, 1458 cm^−1^; HRMS (ESI) *m*/*z* = calcd 586.1462 [M + Na]^+^, found 586.1467 [M + Na]^+^.

#### 3.1.10. Synthesis of 2-(((2R,3S,5R)-5-(6-amino-9H-purin-9-yl)-2-ethynyl-3-hydroxytetrahydrofuran-2-yl) methoxy)-4H-benzo[d][1,3,2]dioxaphosphinine 2-oxide (**5**)

To a stirred solution of phosphoester **18** (156 mg, 0.28 mmol) in MeCN (2.5 mL) and H_2_O (0.5 mL) was added ceric ammonium nitrate (614 mg, 1.1 mmol) at 0 °C. After stirring for 20 min at 0 °C, the resulting mixture was directly purified by silica gel column chromatography to afford **5** (93 mg, 75%) as a white solid. **5**: Colorless crystal; *R*_f_ 0.5 (EtOAc/MeOH = 10/1); ^1^H-NMR (600 MHz, MeOD), δ in ppm major-isomer 8.34 (s, 1H), 8.26 (s, 1H), 7.30–7.26 (1H, overlapped), 7.15–7.11 (1H, overlapped), 7.07–7.06 (m, 1H), 6.96–6.94 (m, 1H), 6.45–6.41 (1H, overlapped), 5.43–5.26 (2H, overlapped), 4.94–4.87 (1H, overlapped), 4.44–4.35 (2H, overlapped), 3.21 (s, 1H), 2.94–2.89 (1H, overlapped), 2.73–2.67 (1H, overlapped); minor-isomer 8.37 (s, 1H), 8.30 (s, 1H), 7.30–7.26 (1H, overlapped), 7.15–7.11 (2H, overlapped), 6.91 (d, *J* = 8.2 Hz, 1H), 6.45-6.41 (1H, overlapped), 5.43–5.26 (2H, overlapped), 4.94–4.87 (1H, overlapped), 4.44–4.35 (2H, overlapped), 3.19 (s, 1H), 2.94–2.89 (1H, overlapped), 2.73-2.67 (1H, overlapped); ^13^C-NMR (150 MHz, MeOD) δ_C_ major-isomer 152.2, 151.0 (d, *J*_C-P_ = 6.5 Hz), 149.4, 145.9, 144.2, 131.0, 126.7, 125.81, 122.0, 120.7, 119.3 (d, *J*_C-P_ = 8.8 Hz), 84.8, 84.41, 80.12, 78.7, 71.5, 70.0 (d, *J*_C-P_ = 6.6 Hz), 38.42; Minor-isomer 152.3, 150.9 (d, *J*_C-P_ = 7.6 Hz), 149.5, 146.0, 144.1, 130.9, 126.8, 125.79, 121.9, 120.6, 119.2 (d, *J*_C-P_ = 7.7 Hz), 84.7, 84.36, 80.06, 78.8, 71.6, 70.2 (d, *J*_C-P_ = 6.5 Hz), 38.37; IR ν_max_ (film) 3246, 3070, 2115, 1688, 1609, 1489 cm^−1^; UV (MeOH) λ_max_ (log ε) 212 (4.35), 261 (3.83) nm; HRMS (ESI) *m*/*z* = calcd 466.0886 [M + Na]^+^, found 466.0889 [M + Na]^+^.

### 3.2. Biological Assays

#### 3.2.1. Cells

MDCK cells and MDCK–SIAT1 cells were cultivated in minimal essential medium (MEM), which was supplemented with 10% heat-inactivated fetal bovine serum from Tissue Culture Biological, Tulare, CA, and 1% penicillin-streptomycin solution from GIBCO-BRL, Rockville, MD.

#### 3.2.2. Virus

As described previously [38], the number of influenza A/Narita/1/09 H1N1pdm viruses were increased in MDCK–SIAT1 cells containing 1% penicillin-streptomycin solution and 5 μg/mL acetylated trypsin at 37 °C in 5% CO_2_ atmosphere for 3 days. The viruses in the culture were harvested, pooled, and clarified before concentration and resuspension in cold phosphate-buffered saline. After aliquoting, aliquots were kept at −80 °C until use.

#### 3.2.3. Cytotoxicity Assay

As described previously [39], each inhibitor was serially diluted in serum-free MEM and transferred onto confluent monolayers of MDCK–SIAT1. After incubation at 37 °C in a 5% CO2 atmosphere for 24 h, the viable cell number was determined with a cell counting kit-8 from DOJINDO Laboratories, Kumamoto, Japan. 

#### 3.2.4. Influenza Growth Inhibition Assay

After two washes with MEM, MDCK–SIAT1 cells were preincubated with an inhibitor at various concentrations in MEM at 37 °C in 5% CO_2_ atmosphere for 1 h. Influenza virus at a multiplicity of infection of 0.03 was pretreated with the same inhibitor at the same concentrations in MEM with 2 μg/mL acetylated trypsin at 4 °C for 1 h. The inhibitor-containing MEM medium on the cells was replaced with the influenza virus-inhibitor mixture. After incubation at 37 °C in a 5% CO_2_ atmosphere for 19–20 h, the medium was removed. Immediately, the infected cells were fixed and permeabilized with methanol. After two washes, the permeabilized cells were stained with primary antiviral NP mouse antibody (4E6) and secondary β-galactosidase-conjugated antimouse IgG antibody. The number of influenza virus-produced nucleoproteins (NPs) in the permeabilized cells in each well was determined by a galactosidase-based fluorescent assay, as previously described [26]. A percentage of the fluorescent intensity of each well relative to the fluorescent intensity of the no-inhibitor control well that contained only the virus was used to express the number of influenza virus-produced NPs in each well. Then, a graph was generated by plotting the percentage of relative numbers of influenza virus-produced NPs against the logarithm of the inhibitor concentrations. After fitting the points on the graph through Prism’s nonlinear regression analysis, the inhibitor concentration that inhibited virus multiplication by 50% in comparison to the control (IC_50_) was obtained. The influenza virus-produced NPs in the permeabilized cells in each well were visualized as a blue color by a peroxidase-based chromogenic assay [40].

## 4. Conclusions

Thus, we developed EdAP, a new 4′-ethynyl-2′-deoxyadenosine 5′-monophosphate analog, as a potent influenza A inhibitor with an IC_50_ of 3.8 μM. EdAP was designed based on the results of the anti-influenza activities of several 4′-ethynyl-adenosine derivatives. The obtained observations could provide helpful information for developing new anti-influenza nucleosides.

## Figures and Tables

**Table 1:** The anti-influenza and cytotoxicity of compounds **1**–**3**.

	H1N1 Influenza A Virus	MDCK–SIAT1
Compound	IC_50_ (μM)	CC_50_^1^ (μM)
**1**	63	>1000
**2**	401	>1000
**3**	>1000	>1000

^1^ The cell counting kit-8 (CCK8) assay was used. IC_50_: 50% inhibitory concentration. CC_50_: 50% cytotoxic concentration. MDCK–SIAT1: Madin–Darby canine kidney cells transfected with human α2-6-sialyltransferase (SIAT1) cDNA.

## Supplementary Materials

The spectra of ^1^H of compounds **5**, **7**, and **9**–**18** and the ^13^C-NMR of compounds **5**, **7**, and **9**–**17** are available online.
