# Supplementary material for: Design, Synthesis, and Biological Evaluation of EdAP, a 4′-Ethynyl-2′-Deoxyadenosine 5′-Monophosphate Analog, as a Potent Influenza a Inhibitor
Source: Molecules. 2019 Jul 17;24(14):2603. doi: 10.3390/molecules24142603 (PMC6681032; doi:10.3390/molecules24142603)
Supplement: Supplementary file 1 [file molecules-24-02603-s001.pdf]

Article

Design, synthesis, and biological evaluation of EdAP, a 4'-ethynyl-2'-deoxyadenosine 5'-monophosphate analog, as a potent influenza A inhibitor

Toshifumi Takeuchi [&1,*], Nongluk Sriwilaijaroen [&2,3], Ayako Sakuraba [1], Ei Hayashi [1], Shinji Kamisuki [4], Yasuo Suzuki [3], Hiroshi Ohrui [5] and Fumio Sugawara [1]

[1] Department of Applied Biological Science, Faculty of Science and Technology, Tokyo University of Science, 2641 Yamazaki, Noda, Chiba 278-8510, Japan.; sugawara@rs.noda.tus.ac.jp (F.S.)
[2] Department of Preclinical Sciences, Faculty of Medicine, Thammasat University, Pathumthani 12120, Thailand; snongluk@hotmail.com (N.S.)
[3] Health Science Hills, College of Life and Health Sciences, Chubu University, Kasugai, Aichi 487-8501, Japan; suzukiy@isc.chubu.ac.jp (Y.S.)
[4] School of Veterinary Medicine, Azabu University, 1-17-71 Fuchinobe, Chuo-ku, Sagamihara, Kanagawa 252-5201, Japan; kamisuki@azabu-u.ac.jp (S.K.)
[5] Yokohama University of Pharmacy, Matano-cho 601, Totsuka-ku, Yokohama, Kanagawa 245-0066, Japan; h.ohrui@hamayaku.ac.jp (H.O.)
[&] These authors contributed equally to this work.
[*] Correspondence: takeuchit@bikaken.or.jp

Supplementary Information

1. Copies of NMR spectra of products

Compound **7**

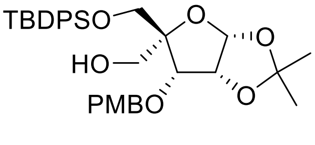
TBDPSO
HO
PMBO

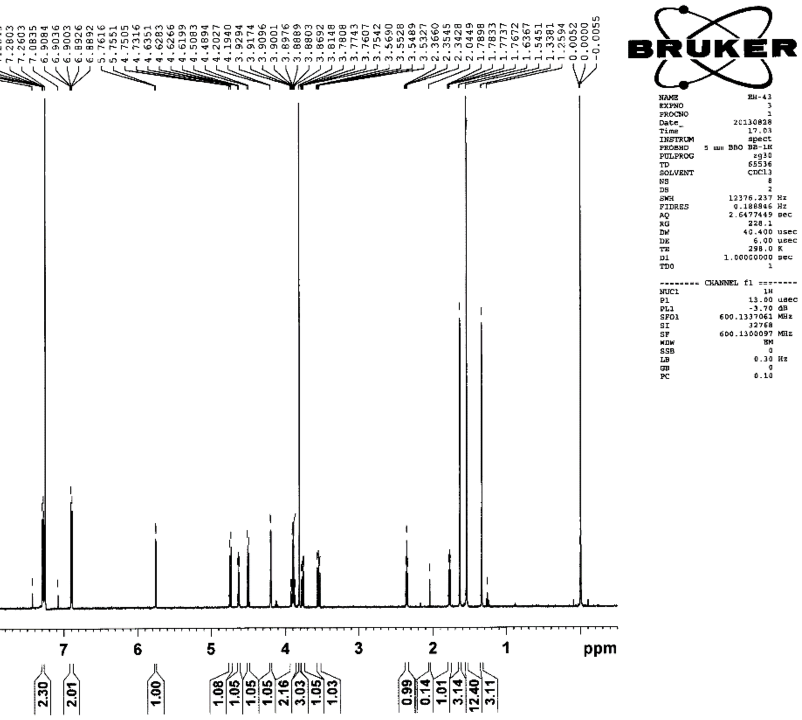
BRUKER
NAME EH-43
EXPNO 3
PROCNO 1
Date_ 20130828
Time 17.03
INSTRUM spect
PROBHD 5 mm BBO BB-1H
PULPROG zg30
TD 65536
SOLVENT CDCl3
NS 8
DS 2
SWH 12376.237 Hz
FIDRES 0.188846 Hz
AQ 2.6477449 sec
RG 228.1
DW 40.400 usec
DE 6.00 usec
TE 298.0 K
D1 1.00000000 sec
TD0 1
======== CHANNEL f1 ========
NUC1 1H
P1 13.00 usec
PL1 -3.70 dB
SFO1 600.1337061 MHz
SI 32768
SF 600.1300097 MHz
WDW EM
SSB 0
LB 0.30 Hz
GB 0
PC 0.10
ppm

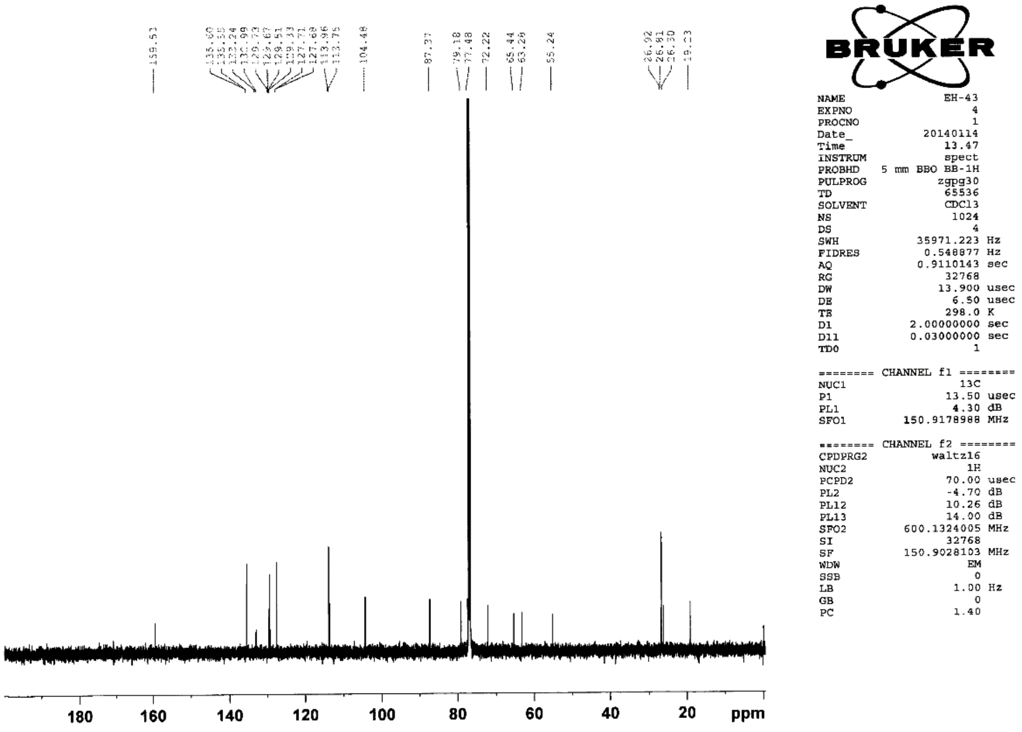
BRUKER
NAME EH-43
EXPNO 4
PROCNO 1
Date_ 20140114
Time 13.47
INSTRUM spect
PROBHD 5 mm BBO BB-1H
PULPROG zgpg30
TD 65536
SOLVENT CDCl3
NS 1024
DS 4
SWH 35971.223 Hz
FIDRES 0.548877 Hz
AQ 0.9110143 sec
RG 32768
DW 13.900 usec
DE 6.50 usec
TE 298.0 K
D1 2.00000000 sec
D11 0.03000000 sec
TD0 1
======== CHANNEL f1 ========
NUC1 13C
P1 13.50 usec
PL1 4.30 dB
SFO1 150.9178988 MHz
======== CHANNEL f2 ========
CPDPRG2 waltz16
NUC2 1H
PCPD2 70.00 usec
PL2 -4.70 dB
PL12 10.26 dB
PL13 14.00 dB
SFO2 600.1324005 MHz
SI 32768
SF 150.9028103 MHz
WDW EM
SSB 0
LB 1.00 Hz
GB 0
PC 1.40
ppm

Compound **9**

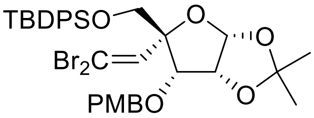

TBDPSO
O
O
Br2C
PMBO
O

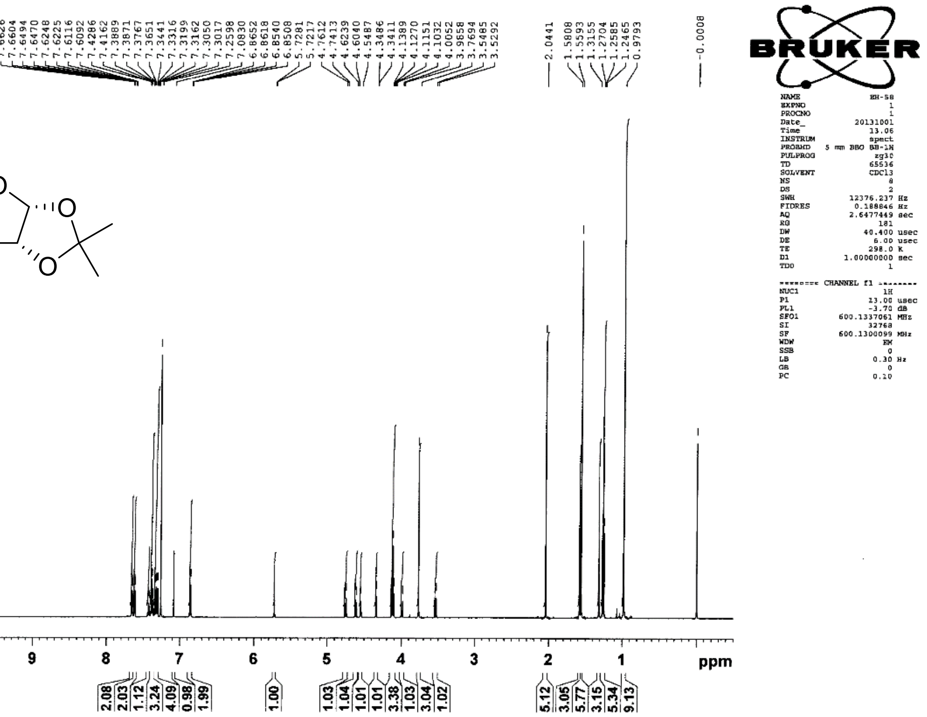

7.6626 7.6604 7.6494 7.6470 7.6248 7.6225 7.6116 7.6092 7.4286 7.4162 7.3889 7.3871 7.3767 7.3651 7.3441 7.3316 7.3199 7.3162 7.3050 7.3017 7.2598 7.0830 6.8652 6.8618 6.8540 6.8508 5.7281 5.7217 4.7612 4.7413 4.6239 4.6040 4.5487 4.3486 4.3411 4.1389 4.1270 4.1151 4.1032 4.0052 3.9858 3.7694 3.5485 3.5292
2.0441 1.5808 1.5593 1.3155 1.2704 1.2585 1.2465 0.9793
-0.0008
BRUKER
NAME EH-58
EXPNO 1
PROCNO 1
Date_ 20131001
Time 13.06
INSTRUM spect
PROBHD 5 mm BBO BB-1H
PULPROG zg30
TD 65536
SOLVENT CDCl3
NS 8
DS 2
SWH 12376.237 Hz
FIDRES 0.188846 Hz
AQ 2.6477449 sec
RG 181
DW 40.400 usec
DE 6.00 usec
TE 298.0 K
D1 1.00000000 sec
TD0 1
======== CHANNEL f1 ========
NUC1 1H
P1 13.00 usec
PL1 -3.70 dB
SFO1 600.1337061 MHz
SI 32768
SF 600.1300099 MHz
WDW EM
SSB 0
LB 0.30 Hz
GB 0
PC 0.10
10 9 8 7 6 5 4 3 2 1 ppm
2.08 2.03 1.12 3.24 4.09 0.98 1.99 1.00 1.03 1.04 1.01 1.01 3.38 1.03 3.04 1.02 5.12 3.05 5.77 3.15 5.34 9.13

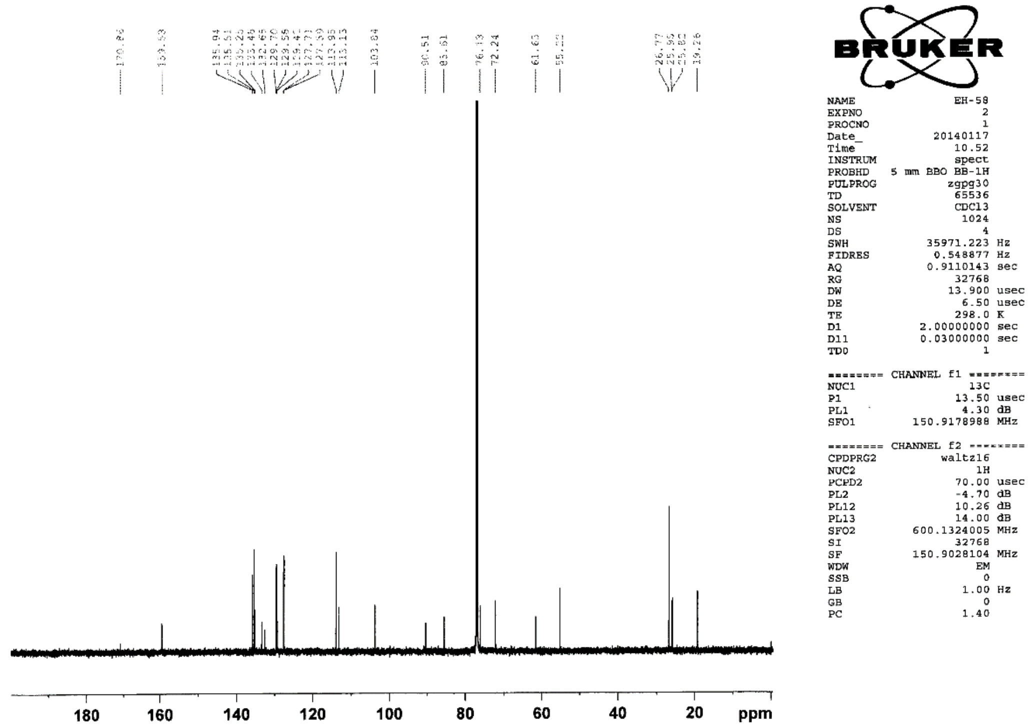

135.94 103.84 90.51 76.13 72.24 61.63 19.26
BRUKER
NAME EH-58
EXPNO 2
PROCNO 1
Date_ 20140117
Time 10.52
INSTRUM spect
PROBHD 5 mm BBO BB-1H
PULPROG zgpg30
TD 65536
SOLVENT CDCl3
NS 1024
DS 4
SWH 35971.223 Hz
FIDRES 0.548877 Hz
AQ 0.9110143 sec
RG 32768
DW 13.900 usec
DE 6.50 usec
TE 298.0 K
D1 2.00000000 sec
D11 0.03000000 sec
TD0 1
======== CHANNEL f1 ========
NUC1 13C
P1 13.50 usec
PL1 4.30 dB
SFO1 150.9178988 MHz
======== CHANNEL f2 ========
CPDPRG2 waltz16
NUC2 1H
PCPD2 70.00 usec
PL2 -4.70 dB
PL12 10.26 dB
PL13 14.00 dB
SFO2 600.1324005 MHz
SI 32768
SF 150.9028104 MHz
WDW EM
SSB 0
LB 1.00 Hz
GB 0
PC 1.40
180 160 140 120 100 80 60 40 20 ppm

Compound **10**

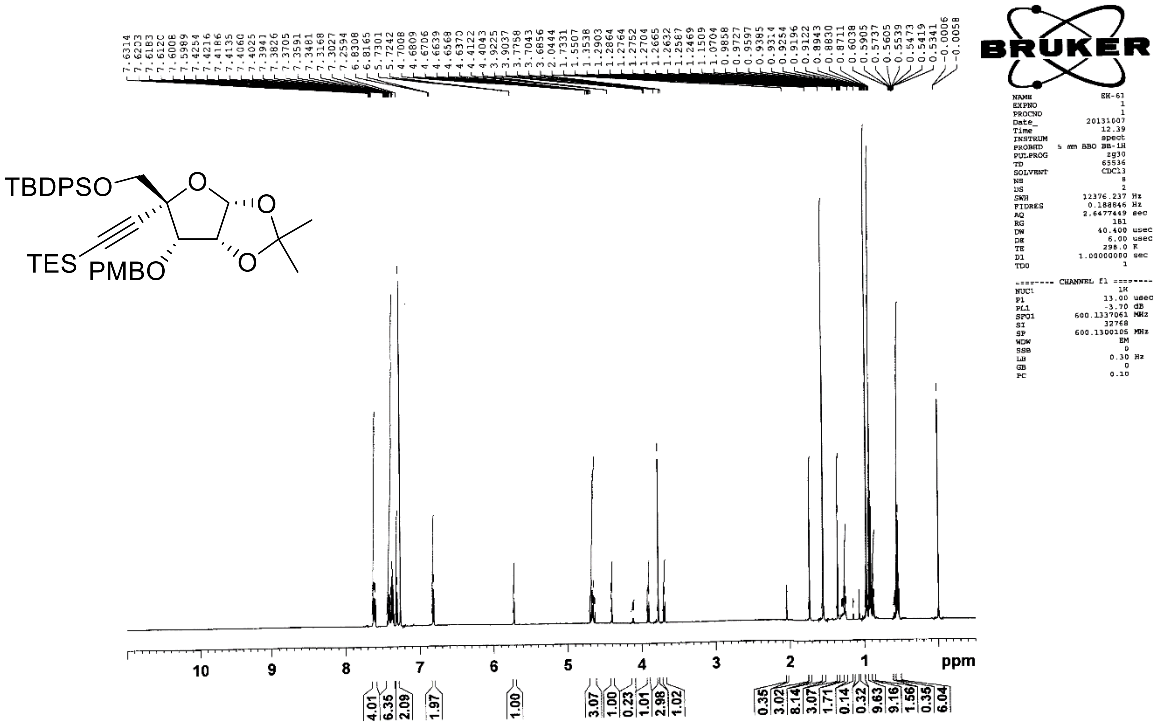
TBDPSO
O
O
O
TES
PMBO
BRUKER
NAME EH-61
EXPNO 1
PROCNO 1
Date_ 20131007
Time 12.39
INSTRUM spect
PROBHD 5 mm BBO BB-1H
PULPROG zg30
TD 65536
SOLVENT CDCl3
NS 8
DS 2
RG 181
TE 298.0 K
D1 1.00000000 sec
TD0 1
CHANNEL f1
NUC1 1H
P1 13.00 usec
PL1 -3.70 dB
SFO1 600.1337061 MHz
SI 32768
SF 600.1300105 MHz
WDW EM
SSB 0
LB 0.30 Hz
GB 0
PC 0.10
10
9
8
7
6
5
4
3
2
1
ppm

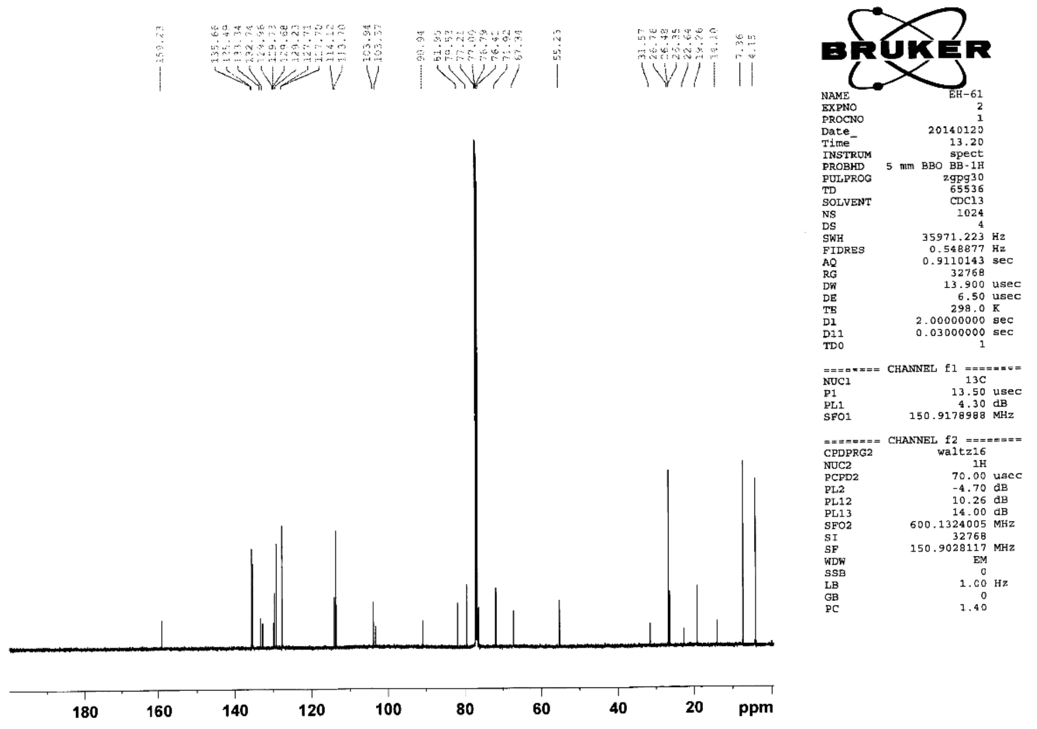
BRUKER
NAME EH-61
EXPNO 2
PROCNO 1
Date_ 20140120
Time 13.20
INSTRUM spect
PROBHD 5 mm BBO BB-1H
PULPROG zgpg30
TD 65536
SOLVENT CDCl3
NS 1024
DS 4
SWH 35971.223 Hz
FIDRES 0.548877 Hz
AQ 0.9110143 sec
RG 32768
DW 13.900 usec
DE 6.50 usec
TE 298.0 K
D1 2.00000000 sec
D11 0.03000000 sec
TD0 1
CHANNEL f1
NUC1 13C
P1 13.50 usec
PL1 4.30 dB
SFO1 150.9178988 MHz
CHANNEL f2
CPDPRG2 waltz16
NUC2 1H
PCPD2 70.00 usec
PL2 -4.70 dB
PL12 10.26 dB
PL13 14.00 dB
SFO2 600.1324005 MHz
SI 32768
SF 150.9028117 MHz
WDW EM
SSB 0
LB 1.00 Hz
GB 0
PC 1.40
180
160
140
120
100
80
60
40
20
ppm

Compound **11**

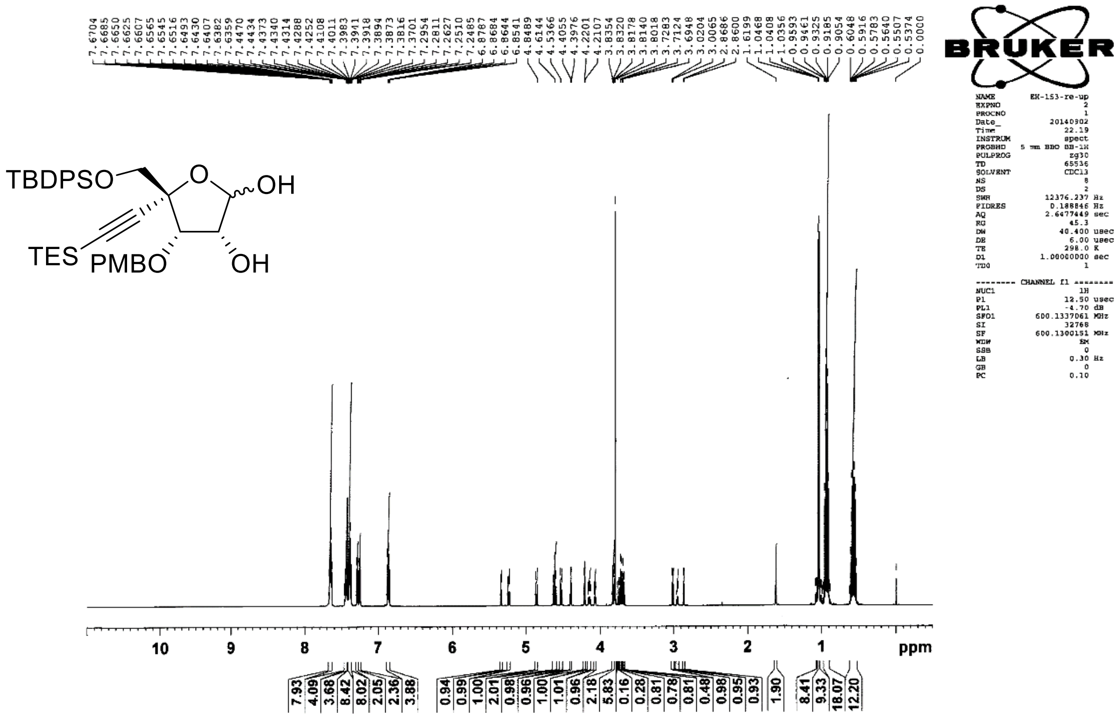

TBDPSO
O
OH
TES
PMBO
OH
7.6704 7.6685 7.6650 7.6625 7.6607 7.6565 7.6545 7.6516 7.6493 7.6430 7.6407 7.6382 7.6359 7.4470 7.4434 7.4373 7.4340 7.4314 7.4288 7.4252 7.4108 7.4011 7.3983 7.3941 7.3918 7.3894 7.3873 7.3816 7.3701 7.2954 7.2811 7.2627 7.2510 7.2485 6.8787 6.8684 6.8644 6.8541 4.8489 4.6144 4.5366 4.4055 4.3976 4.2201 4.2107 3.8354 3.8320 3.8178 3.8140 3.8018 3.7283 3.7124 3.6948 3.0204 3.0065 2.8686 2.8600 1.6199 1.0468 1.0408 1.0356 0.9593 0.9461 0.9325 0.9185 0.9054 0.6048 0.5916 0.5783 0.5640 0.5507 0.5374 0.0000
BRUKER
10 9 8 7 6 5 4 3 2 1 ppm
7.93 4.09 3.68 8.42 8.02 2.05 2.36 3.88 0.94 0.99 1.00 2.01 0.98 0.96 1.00 1.01 0.96 2.18 5.83 0.16 0.28 0.81 0.78 0.81 0.48 0.98 0.98 0.95 0.93 1.90 8.41 9.33 18.07 12.20

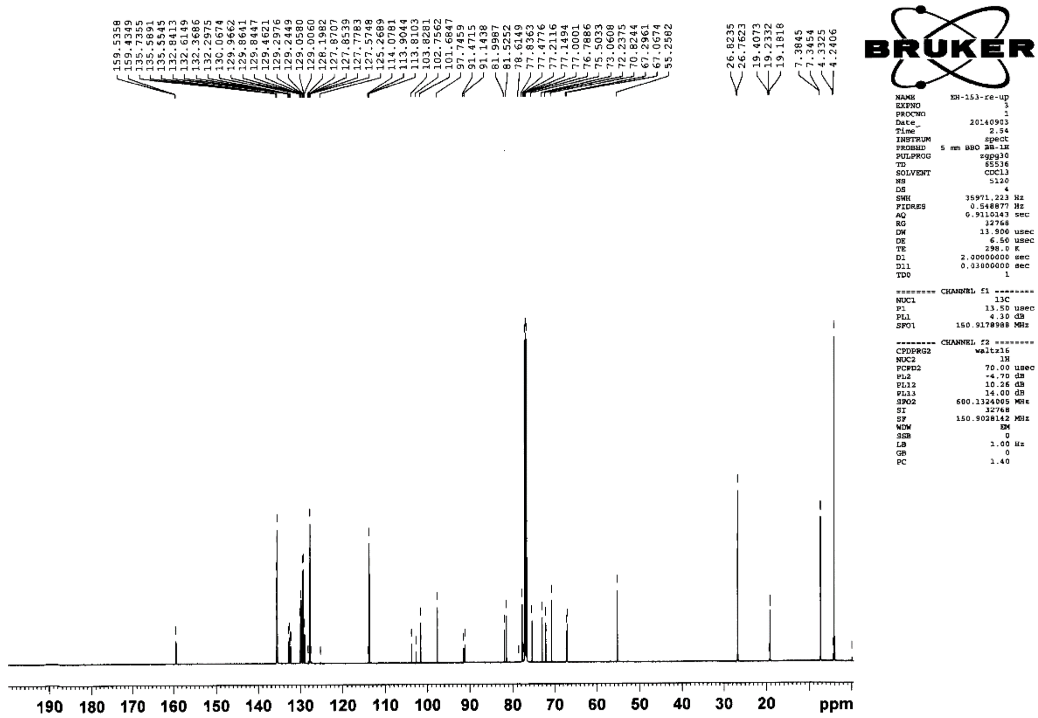

159.5358 159.4349 135.7355 135.5891 135.5545 132.8413 132.6149 132.3686 132.2975 130.0674 129.9662 129.9641 129.8447 129.4621 129.2976 129.2449 129.0580 129.0060 128.1982 127.8707 127.8539 127.7783 127.5748 125.2689 114.0781 113.9044 113.8103 103.8281 102.7562 101.6847 97.7659 91.4715 91.1438 81.9987 81.5252 78.6149 77.8363 77.4776 77.2116 77.1494 77.0001 76.7886 75.5033 73.0608 72.2375 70.8244 67.2961 67.0674 55.2582 26.8235 26.7623 19.4073 19.2332 19.1818 7.3845 7.3454 4.3325 4.2406
BRUKER
190 180 170 160 150 140 130 120 110 100 90 80 70 60 50 40 30 20 ppm

Compound **13**

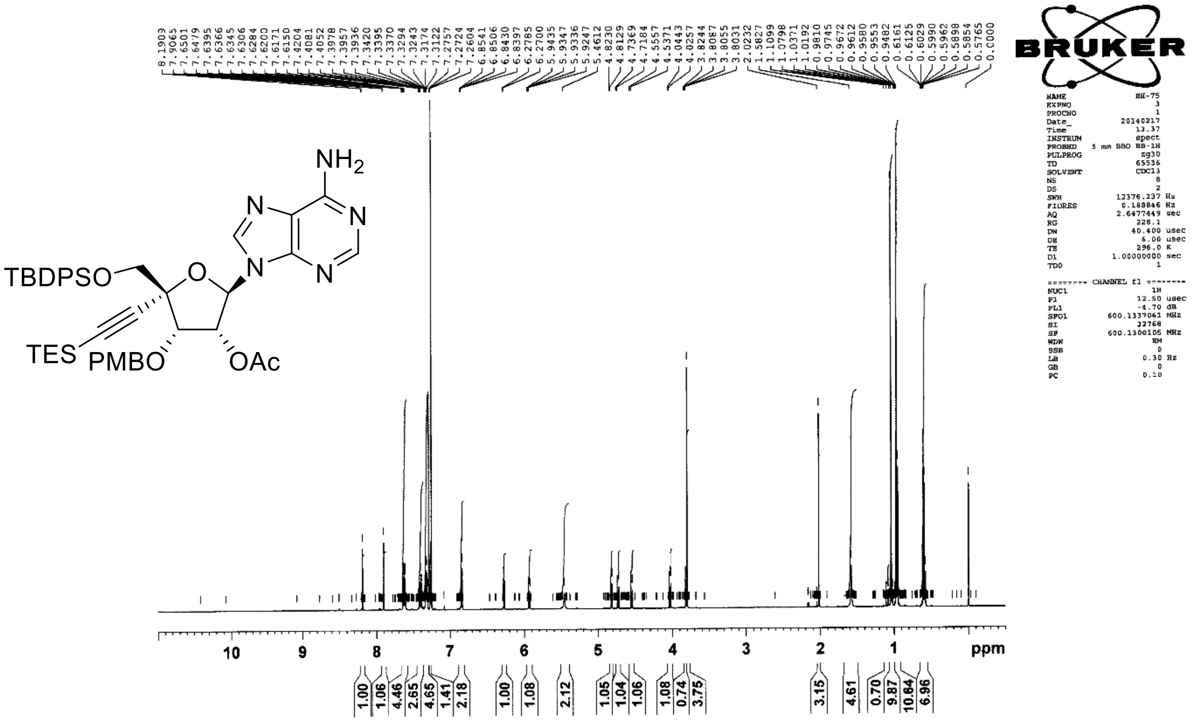

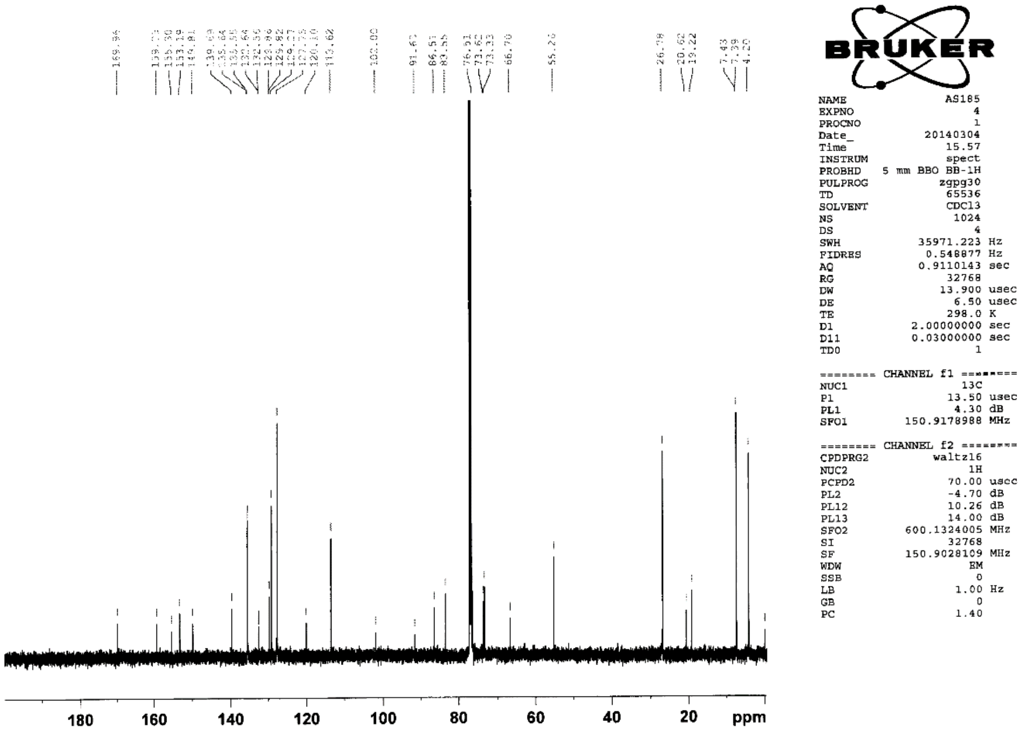

Compound **14**

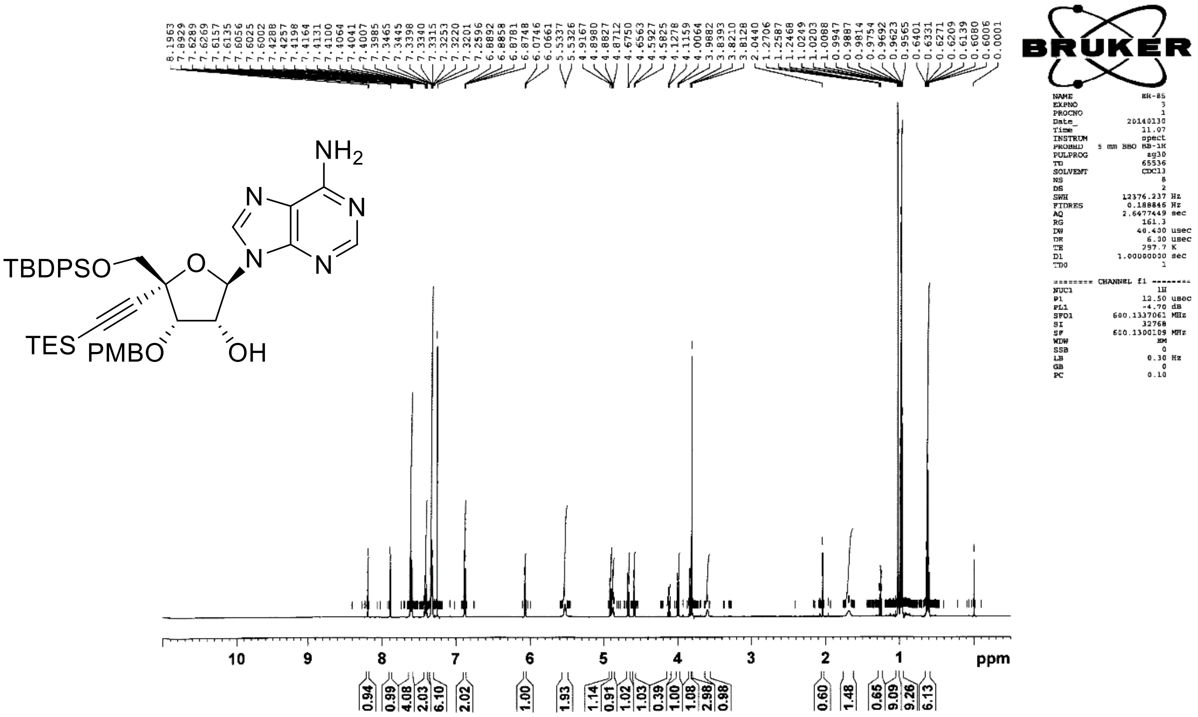
NH2
N
N
N
N
O
TBDPSO
TES
PMBO
OH
8.1963
7.8929
7.6289
7.6269
7.6157
7.6135
7.6036
7.6025
7.6002
7.4288
7.4257
7.4198
7.4164
7.4131
7.4100
7.4064
7.4041
7.4007
7.3985
7.3465
7.3445
7.3398
7.3340
7.3315
7.3253
7.3220
7.3201
7.2596
6.8892
6.8858
6.8781
6.8748
6.0746
6.0661
5.5337
5.5326
4.9167
4.8980
4.8827
4.8712
4.6750
4.6563
4.5927
4.5825
4.1278
4.1159
4.0064
3.9882
3.8393
3.8210
3.8128
2.0440
1.2706
1.2587
1.2468
1.0249
1.0203
1.0088
0.9947
0.9887
0.9814
0.9754
0.9692
0.9623
0.9565
0.6401
0.6331
0.6271
0.6209
0.6139
0.6080
0.6006
0.0001
BRUKER
NAME EH-85
EXPNO 3
PROCNO 1
Date_ 20140130
Time 11.07
INSTRUM spect
PROBHD 5 mm BBO BB-1H
PULPROG zg30
TD 65536
SOLVENT CDCl3
NS 8
DS 2
SWH 12376.237 Hz
FIDRES 0.188846 Hz
AQ 2.6477449 sec
RG 161.3
DW 40.400 usec
DE 6.00 usec
TE 297.7 K
D1 1.00000000 sec
TD0 1
======== CHANNEL f1 ========
NUC1 1H
P1 12.50 usec
PL1 -4.70 dB
SFO1 600.1337061 MHz
SI 32768
SF 600.1300109 MHz
WDW EM
SSB 0
LB 0.30 Hz
GB 0
PC 0.10
10
9
8
7
6
5
4
3
2
1
ppm
0.94
0.99
4.08
2.03
6.10
2.02
1.00
1.93
1.14
0.91
1.02
1.03
0.39
1.00
1.08
2.98
0.98
0.60
1.48
0.65
9.09
9.26
6.13

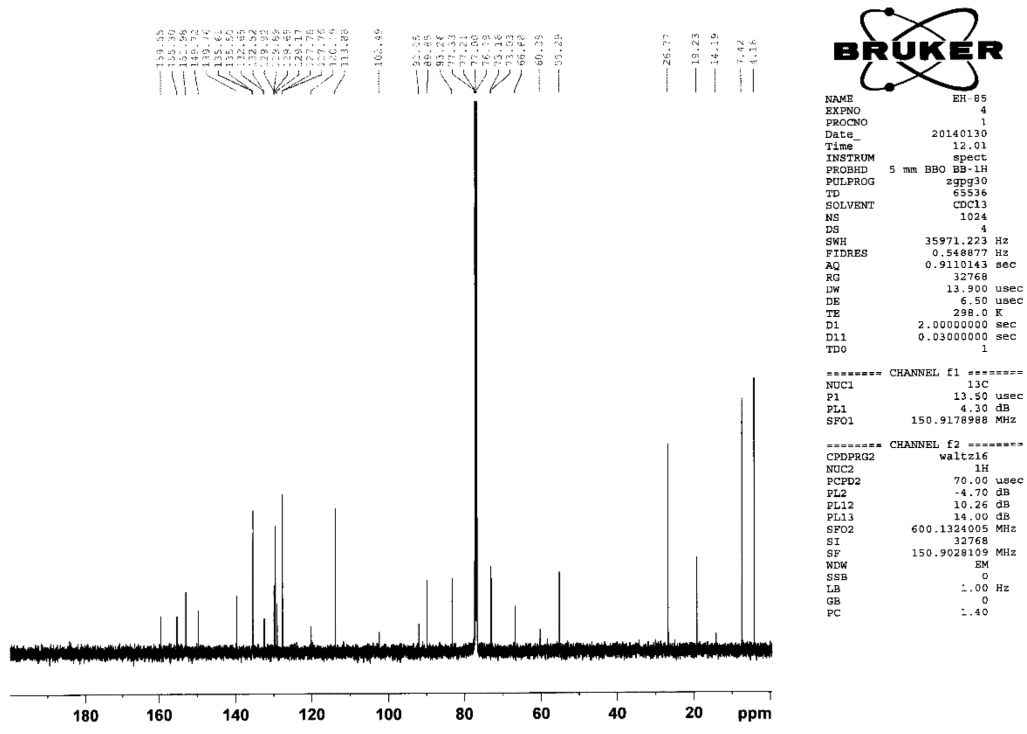
BRUKER
NAME EH-85
EXPNO 4
PROCNO 1
Date_ 20140130
Time 12.01
INSTRUM spect
PROBHD 5 mm BBO BB-1H
PULPROG zgpg30
TD 65536
SOLVENT CDCl3
NS 1024
DS 4
SWH 35971.223 Hz
FIDRES 0.548877 Hz
AQ 0.9110143 sec
RG 32768
DW 13.900 usec
DE 6.50 usec
TE 298.0 K
D1 2.00000000 sec
D11 0.03000000 sec
TD0 1
======== CHANNEL f1 ========
NUC1 13C
P1 13.50 usec
PL1 4.30 dB
SFO1 150.9178988 MHz
======== CHANNEL f2 ========
CPDPRG2 waltz16
NUC2 1H
PCPD2 70.00 usec
PL2 -4.70 dB
PL12 10.26 dB
PL13 14.00 dB
SFO2 600.1324005 MHz
SI 32768
SF 150.9028109 MHz
WDW EM
SSB 0
LB 1.00 Hz
GB 0
PC 1.40
180
160
140
120
100
80
60
40
20
ppm

Compound **15**

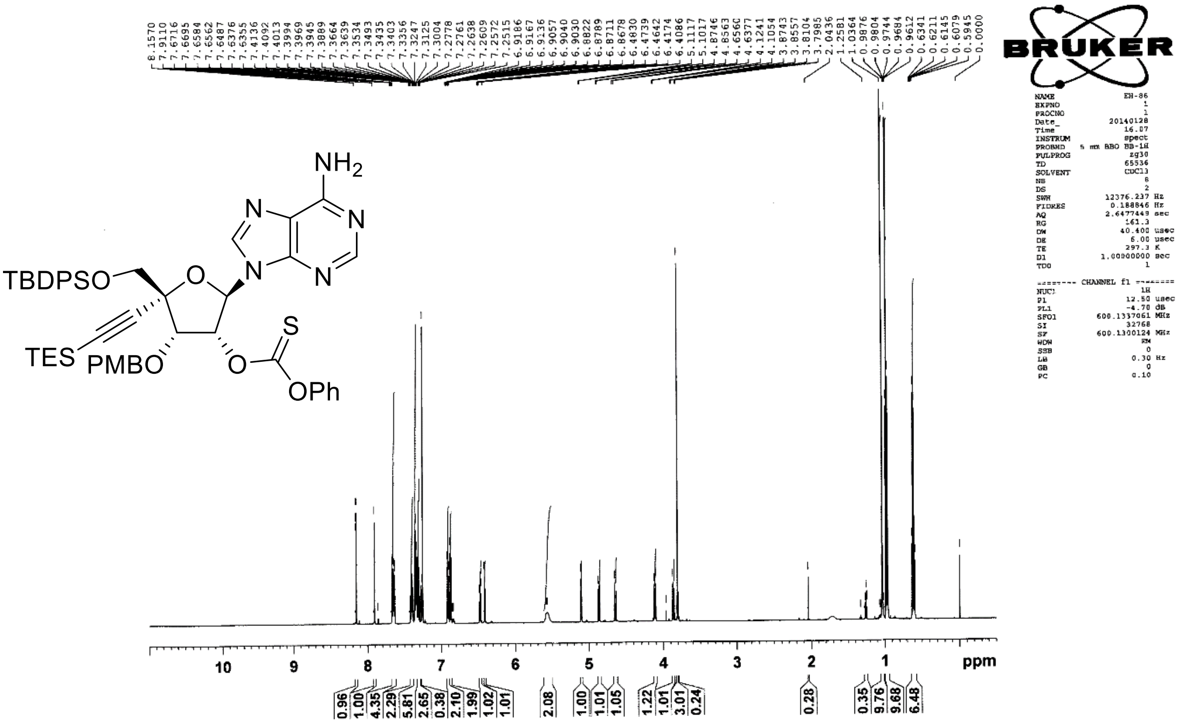
NH2
N
N
N
N
O
TBDPSO
TES
PMBO
O
S
OPh
BRUKER
ppm
10
9
8
7
6
5
4
3
2
1

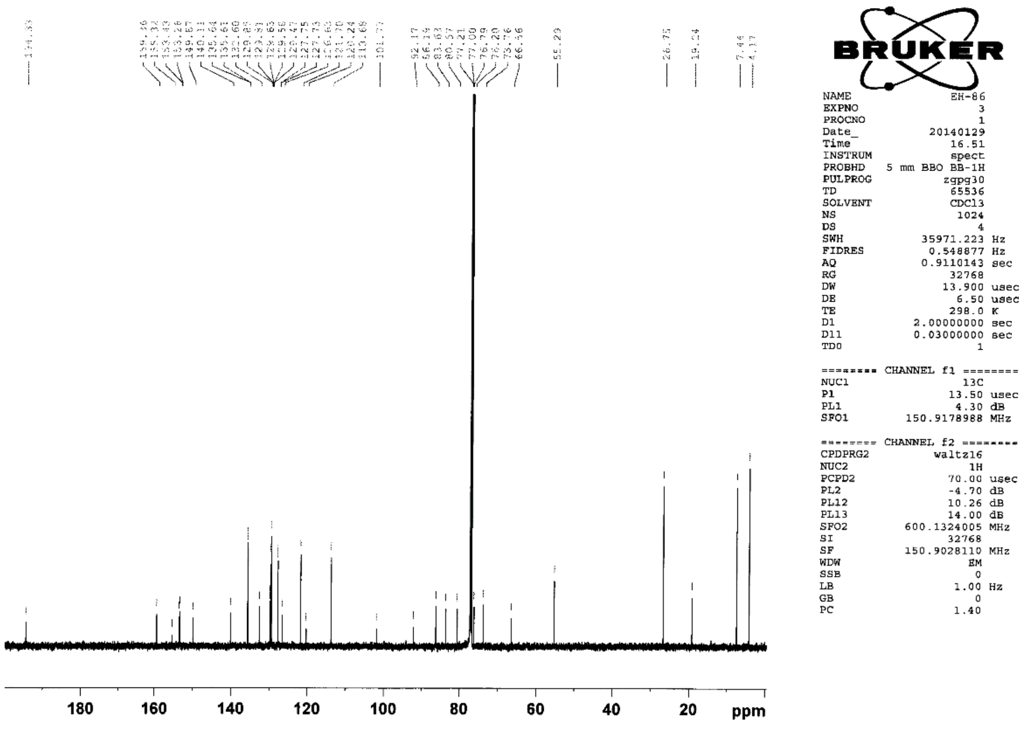
BRUKER
NAME EH-86
EXPNO 3
PROCNO 1
Date_ 20140129
Time 16.51
INSTRUM spect
PROBHD 5 mm BBO BB-1H
PULPROG zgpg30
TD 65536
SOLVENT CDCl3
NS 1024
DS 4
SWH 35971.223 Hz
FIDRES 0.548877 Hz
AQ 0.9110143 sec
RG 32768
DW 13.900 usec
DE 6.50 usec
TE 298.0 K
D1 2.00000000 sec
D11 0.03000000 sec
TD0 1
======== CHANNEL f1 ========
NUC1 13C
P1 13.50 usec
PL1 4.30 dB
SFO1 150.9178988 MHz
======== CHANNEL f2 ========
CPDPRG2 waltz16
NUC2 1H
PCPD2 70.00 usec
PL2 -4.70 dB
PL12 10.26 dB
PL13 14.00 dB
SFO2 600.1324005 MHz
SI 32768
SF 150.9028110 MHz
WDW EM
SSB 0
LB 1.00 Hz
GB 0
PC 1.40
ppm
180
160
140
120
100
80
60
40
20

Compound **16**

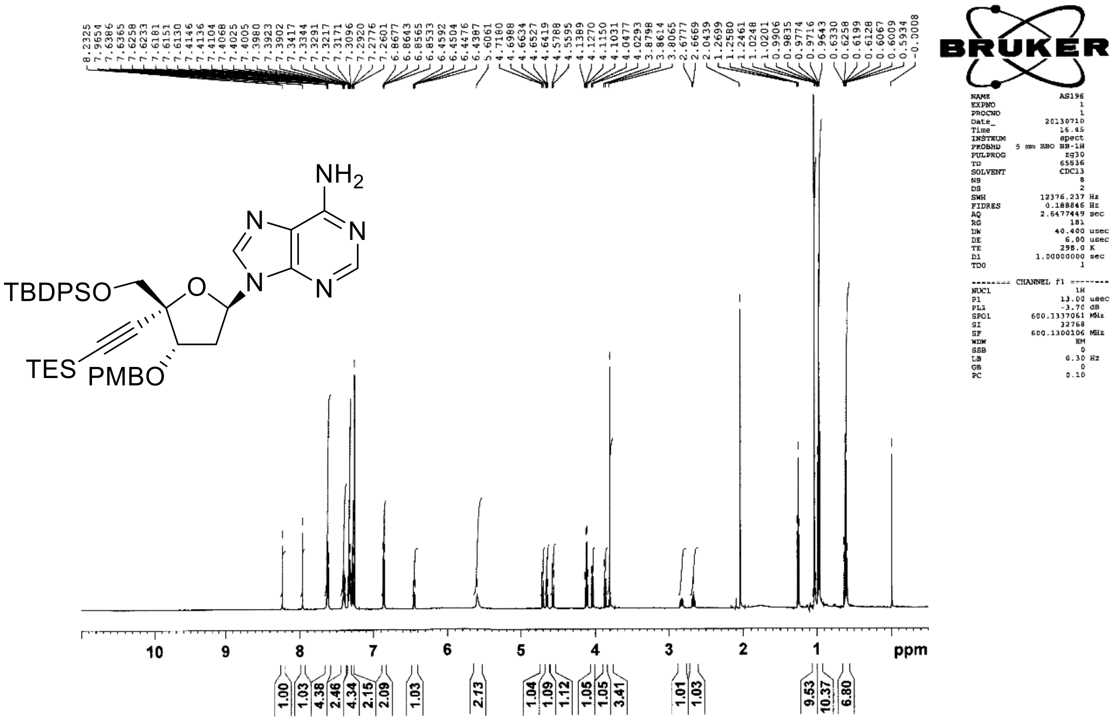

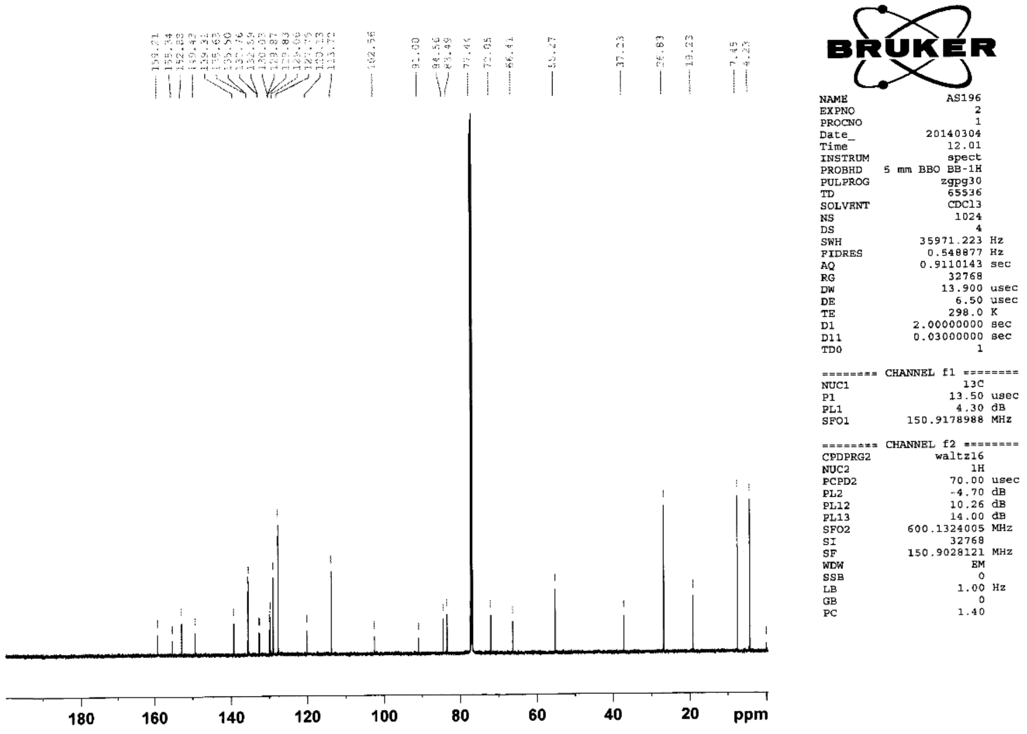

Compound **17**

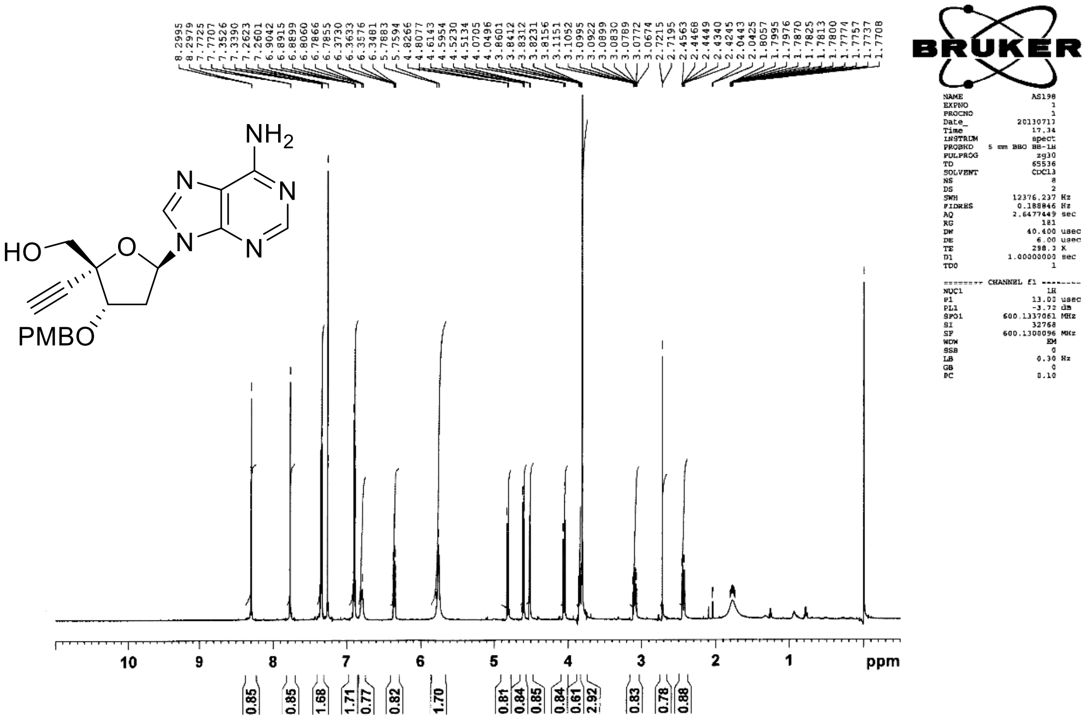

8.2995
8.2979
7.7725
7.7707
7.3526
7.3390
7.2623
7.2601
6.9042
6.8915
6.8899
6.8060
6.7866
6.7855
6.3730
6.3633
6.3576
6.3481
5.7883
5.7594
4.8266
4.8077
4.6143
4.5954
4.5230
4.5134
4.0705
4.0496
3.8601
3.8412
3.8312
3.8231
3.8156
3.1151
3.1052
3.0995
3.0922
3.0899
3.0830
3.0789
3.0772
3.0674
2.7215
2.7195
2.4563
2.4468
2.4449
2.4340
2.4245
2.0443
2.0425
1.8057
1.7995
1.7976
1.7870
1.7825
1.7813
1.7800
1.7774
1.7757
1.7737
1.7708
NH2
N
N
N
N
HO
O
PMBO
NAME AS198
EXPNO 1
PROCNO 1
Date_ 20130717
Time 17.34
INSTRUM spect
PROBHD 5 mm BBO BB-1H
PULPROG zg30
TD 65536
SOLVENT CDCl3
NS 8
DS 2
SWH 12376.237 Hz
FIDRES 0.188846 Hz
AQ 2.6477449 sec
RG 181
DW 40.400 usec
DE 6.00 usec
TE 298.1 K
D1 1.00000000 sec
TD0 1
======== CHANNEL f1 ========
NUC1 1H
P1 13.00 usec
PL1 -3.70 dB
SFO1 600.1337061 MHz
SI 32768
SF 600.1300096 MHz
WDW EM
SSB 0
LB 0.30 Hz
GB 0
PC 0.10
10
9
8
7
6
5
4
3
2
1
ppm
0.85
0.85
1.68
1.71
0.77
0.82
1.70
0.81
0.84
0.85
0.84
0.61
2.92
0.83
0.78
0.88

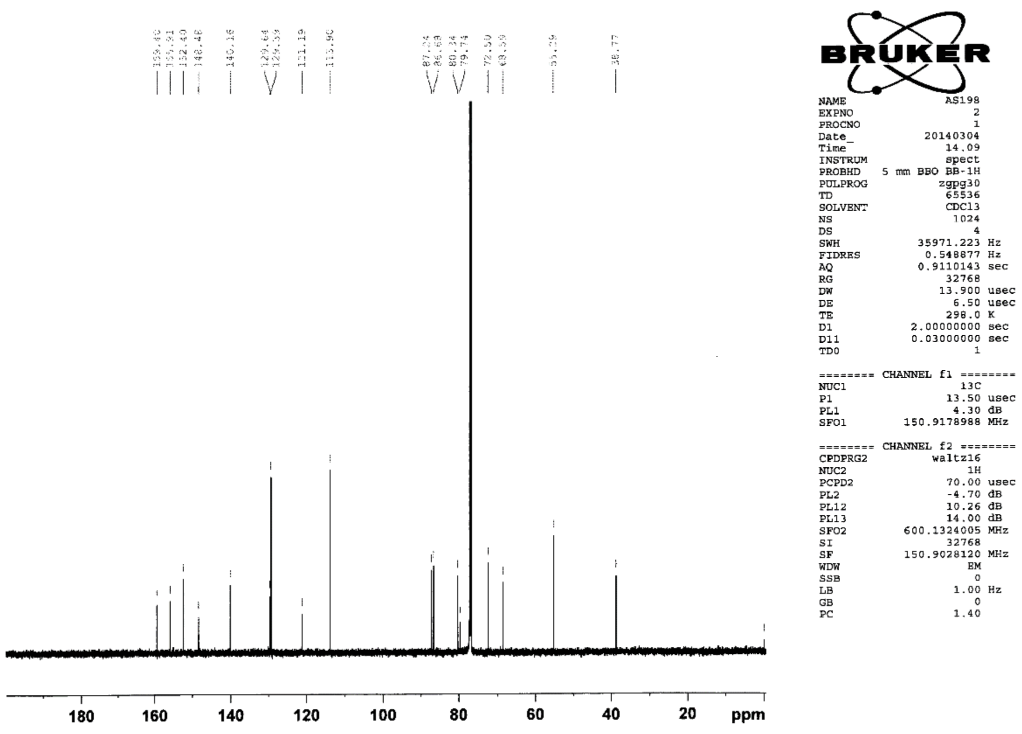

140.16
121.19
113.90
80.34
79.74
72.50
38.77
NAME AS198
EXPNO 2
PROCNO 1
Date_ 20140304
Time 14.09
INSTRUM spect
PROBHD 5 mm BBO BB-1H
PULPROG zgpg30
TD 65536
SOLVENT CDCl3
NS 1024
DS 4
SWH 35971.223 Hz
FIDRES 0.548877 Hz
AQ 0.9110143 sec
RG 32768
DW 13.900 usec
DE 6.50 usec
TE 298.0 K
D1 2.00000000 sec
D11 0.03000000 sec
TD0 1
======== CHANNEL f1 ========
NUC1 13C
P1 13.50 usec
PL1 4.30 dB
SFO1 150.9178988 MHz
======== CHANNEL f2 ========
CPDPRG2 waltz16
NUC2 1H
PCPD2 70.00 usec
PL2 -4.70 dB
PL12 10.26 dB
PL13 14.00 dB
SFO2 600.1324005 MHz
SI 32768
SF 150.9028120 MHz
WDW EM
SSB 0
LB 1.00 Hz
GB 0
PC 1.40
180
160
140
120
100
80
60
40
20
ppm

Compound **18**

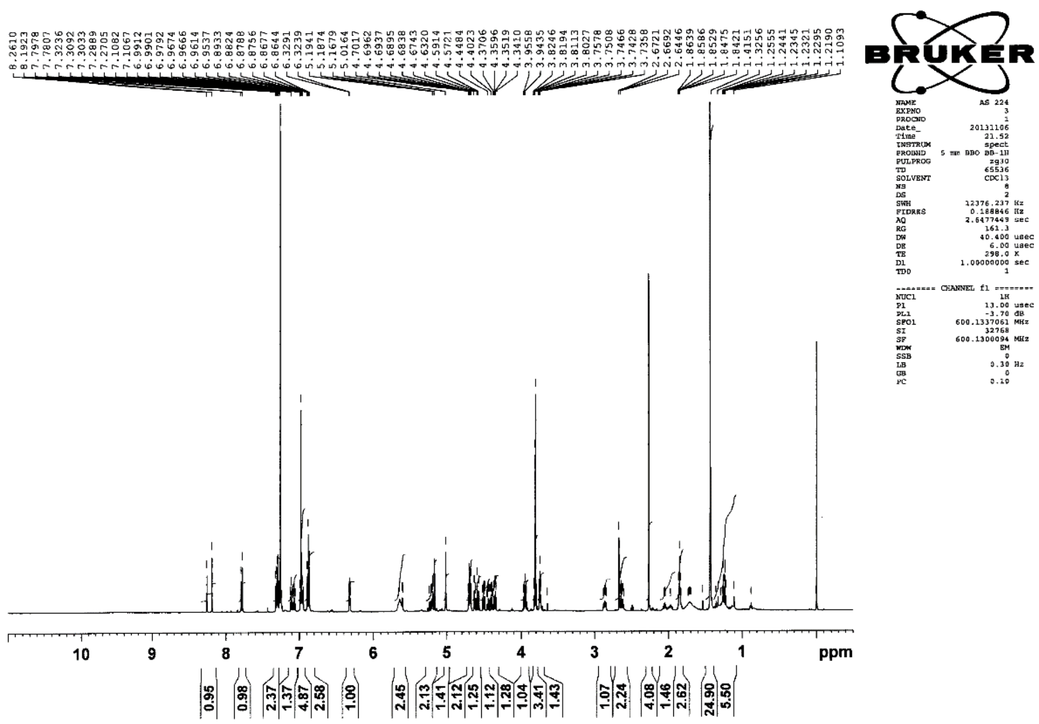

Compound **5**

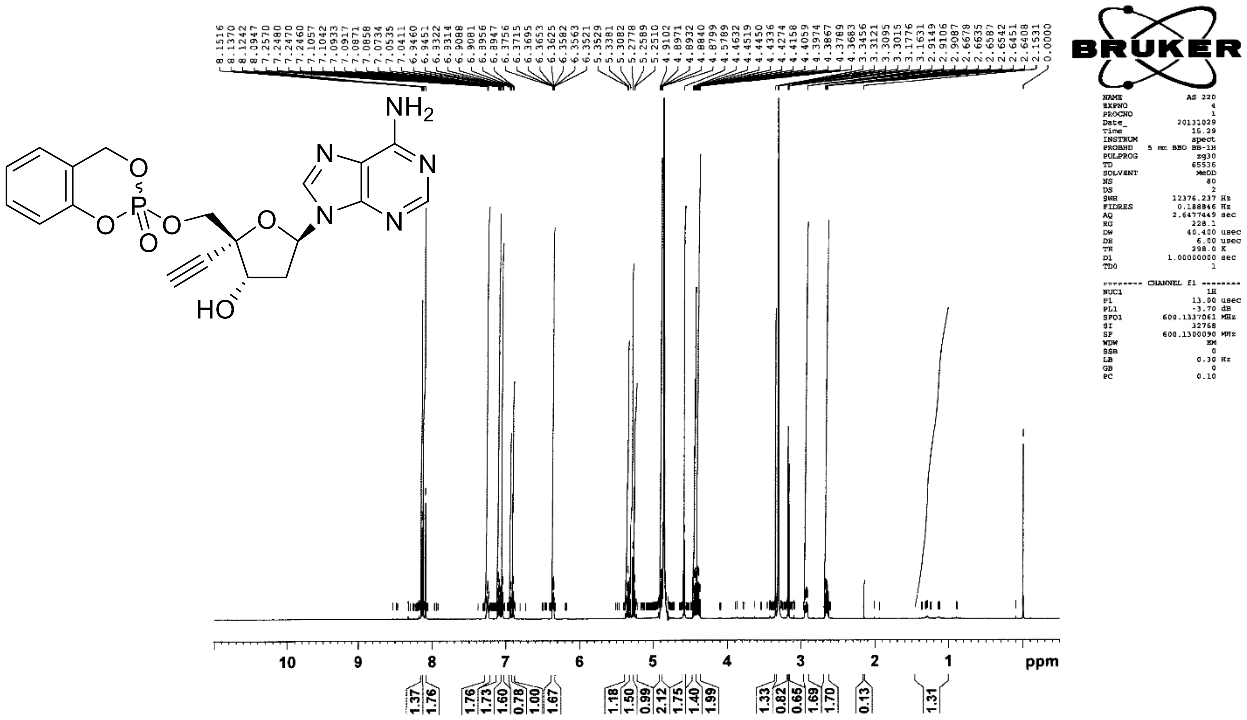

NH2
N
O
P
HO
BRUKER
10
9
8
7
6
5
4
3
2
1
ppm

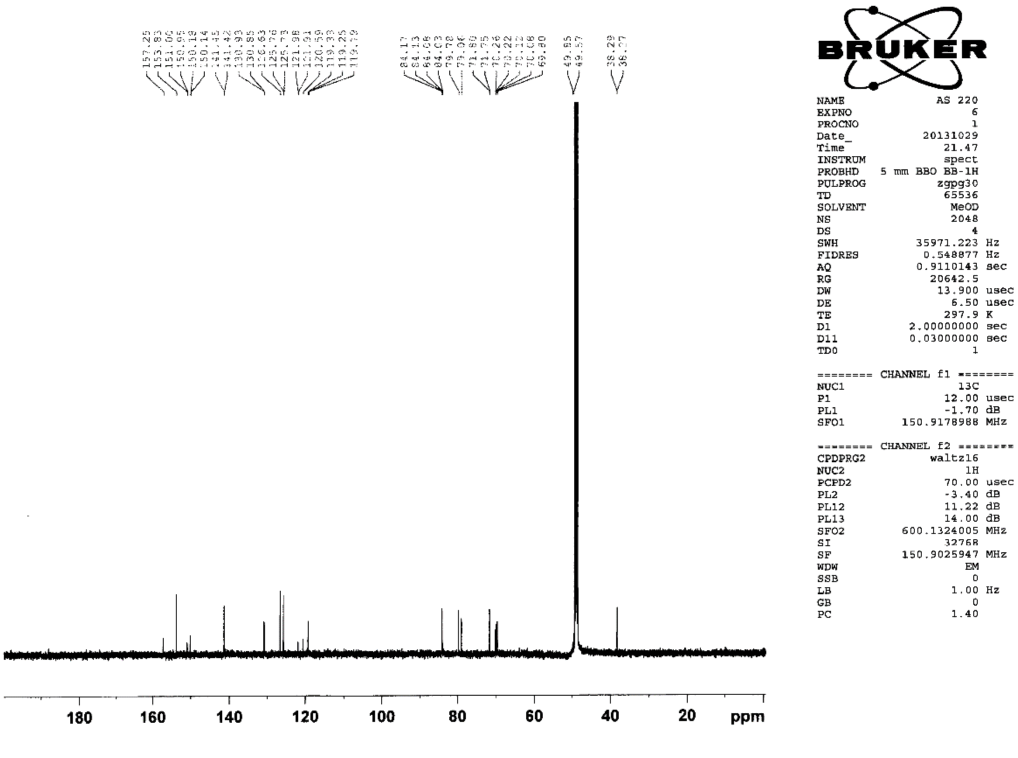

BRUKER
NAME AS 220
EXPNO 6
PROCNO 1
Date_ 20131029
Time 21.47
INSTRUM spect
PROBHD 5 mm BBO BB-1H
PULPROG zgpg30
TD 65536
SOLVENT MeOD
NS 2048
DS 4
SWH 35971.223 Hz
FIDRES 0.548877 Hz
AQ 0.9110143 sec
RG 20642.5
DW 13.900 usec
DE 6.50 usec
TE 297.9 K
D1 2.00000000 sec
D11 0.03000000 sec
TD0 1
======== CHANNEL f1 ========
NUC1 13C
P1 12.00 usec
PL1 -1.70 dB
SFO1 150.9178988 MHz
======== CHANNEL f2 ========
CPDPRG2 waltz16
NUC2 1H
PCPD2 70.00 usec
PL2 -3.40 dB
PL12 11.22 dB
PL13 14.00 dB
SFO2 600.1324005 MHz
SI 32768
SF 150.9025947 MHz
WDW EM
SSB 0
LB 1.00 Hz
GB 0
PC 1.40
180
160
140
120
100
80
60
40
20
ppm
